# DNA repair factor BRCA1 depletion occurs in Alzheimer brains and impairs cognitive function in mice

**DOI:** 10.1038/ncomms9897

**Published:** 2015-11-30

**Authors:** Elsa Suberbielle, Biljana Djukic, Mark Evans, Daniel H. Kim, Praveen Taneja, Xin Wang, Mariel Finucane, Joseph Knox, Kaitlyn Ho, Nino Devidze, Eliezer Masliah, Lennart Mucke

**Affiliations:** 1 Gladstone Institute of Neurological Disease, San Francisco, California 94158, USA; 2 Department of Neurology, University of California, San Francisco, San Francisco, California 94158, USA; 3 Gladstone Institute of Cardiovascular Disease, San Francisco, California 94158, USA; 4 Department of Neuroscience, University of California, San Diego, La Jolla, California 92093, USA; 5 Department of Pathology, University of California, San Diego, La Jolla, California 92093, USA

## Abstract

Maintaining DNA integrity is vital for all cells and organisms. Defective DNA repair may contribute to neurological disorders, including Alzheimer's disease (AD). We found reduced levels of BRCA1, but not of other DNA repair factors, in the brains of AD patients and human amyloid precursor protein (hAPP) transgenic mice. Amyloid-β oligomers reduced BRCA1 levels in primary neuronal cultures. In wild-type mice, knocking down neuronal BRCA1 in the dentate gyrus caused increased DNA double-strand breaks, neuronal shrinkage, synaptic plasticity impairments, and learning and memory deficits, but not apoptosis. Low levels of hAPP/Amyloid-β overexpression exacerbated these effects. Physiological neuronal activation increased BRCA1 levels, whereas stimulating predominantly extrasynaptic *N*-methyl-D-aspartate receptors promoted the proteasomal degradation of BRCA1. We conclude that BRCA1 is regulated by neuronal activity, protects the neuronal genome, and critically supports neuronal integrity and cognitive functions. Pathological accumulation of Aβ depletes neuronal BRCA1, which may contribute to cognitive deficits in AD.

## Abstract (web summary)

DNA repair deficits have been suggested to play a role in Alzheimer's pathology. Here, the authors report reduced levels of the DNA repair factor BRCA1 in patient brains, and provide evidence that loss of BRCA1 in the dentate gyrus leads to spatial learning and memory deficits in mice.

## Graphical abstract

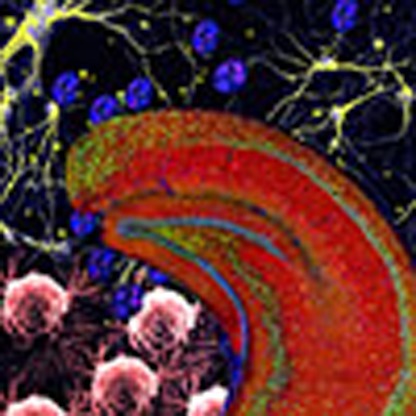

Effective repair of DNA damage is essential for the survival of cells and most individual organisms and species. Ineffective repair can result in cell death, cancer and neurological disease^1^,^2^. Alzheimer's disease (AD) and other neurodegenerative disorders are associated with excessive neuronal DNA damage^3^.

We have shown that physiological increases in brain activity transiently increase neuronal DNA double-strand breaks (DSBs) without causing neuronal apoptosis^4^. In wild-type (WT) mice, the DSBs were rapidly repaired after neuronal activity returned to baseline. Human amyloid precursor protein (hAPP) transgenic mice from line J20, which simulate key aspects of AD^5^, had higher levels of neuronal DSBs at baseline and abnormal persistence of activity-induced neuronal DSBs^4^. In principle, these alterations could result from excessive formation or defective repair of DSBs. Because DNA repair is defective in several neurodegenerative diseases^1^,^2^, we hypothesized that the abnormal accumulation of DSBs in neurons of hAPP mice is caused by alterations in their DNA repair machinery.

We show that the levels of a specific DNA repair factor are decreased in brains of hAPP-J20 mice and of patients with AD. Knocking down this factor in the dentate gyrus (DG) of mice increased neuronal DSB levels in this brain region and caused behavioural deficits without causing neuronal loss.

## Results

### BRCA1 depletion in hAPP brains and in neurons exposed to Aβ

**Figure 1:**
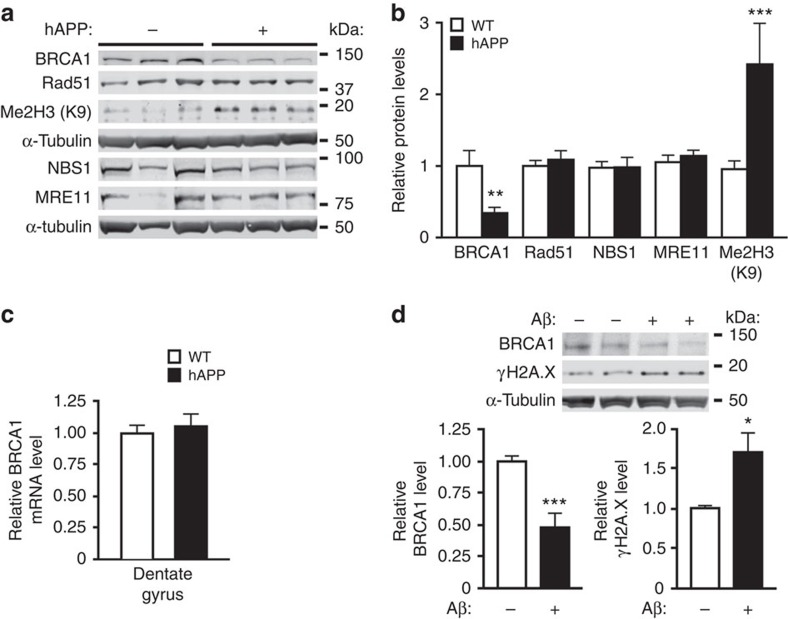
BRCA1 reduction in brain tissues from hAPP-J20 mice and in primary neurons exposed to Aβ oligomers. (**a**,**b**) Levels of DNA repair factors BRCA1, Rad51, NBS1 and MRE11, and of histone 3 dimethylated on lysine 9 (Me2H3(K9)) in the DG of WT and hAPP-J20 mice. (**a**) Representative western blot. (**b**) Quantitation of western blot signals. The average DNA-repair factor or histone to α-tubulin ratio in WT mice was arbitrarily defined as 1.0. *n*=17–20 mice per genotype for BRCA1 and *n*=12–14 mice per genotype for the other proteins. Age, 4–8 months. (**c**) Levels of BRCA1 mRNA in the DG were assessed by RT–qPCR. The average BRCA1 to GAPDH mRNA ratio in WT mice was arbitrarily defined as 1.0. *n*=11–13 mice per genotype. Age, 4–6 months. (**d**) Cultures of primary hippocampal neurons from WT mice were exposed to Aβ oligomers (1 μM) (+) or vehicle (–) for 5 h. Levels of BRCA1 and the DSB marker γH2A.X were determined by western blotting. The average DNA-repair factor to α-tubulin ratio in vehicle-treated cultures was defined as 1.0. *n*=6–8 wells per condition from three independent experiments. In western blots each lane contained a sample from a different mouse (**a**) or culture well (**d**). *P<0.05, **P<0.01, ***P<0.001 versus WT (**b**) or vehicle (**d**) by *t*-test (with Welch correction in **d**). Bars represent means±s.e.m.

We compared the levels of key components of the DSB repair machinery in hAPP-J20 mice and WT controls. In DG, where neuronal DSBs and other biochemical alterations differ most between these groups^4^,^6^,^7^, hAPP-J20 mice had a selective, almost 70% reduction in breast cancer factor 1 (BRCA1), whereas MRE11, NBS1 and RAD51 levels were unaffected (Fig. 1a,b). In parietal cortex, BRCA1 levels were decreased by 45% in hAPP-J20 mice (Supplementary Fig. 1a,b). hAPP-J20 mice had normal levels of BRCA1 mRNA in the DG and parietal cortex (Fig. 1c and Supplementary Fig. 1c), suggesting post-transcriptional depletion of BRCA1 protein.

Aβ_1–42_ oligomers, the most likely mediators of Aβ-dependent neuronal dysfunction^8^, reduced BRCA1 levels in primary neuronal cultures by 50% and increased levels of the DSB marker γH2A.X by 70% (Fig. 1d). Thus, high levels of soluble Aβ assemblies, which also occur in brains of hAPP mice and AD patients^8^, can reduce neuronal BRCA1 levels.

BRCA1 is a 208-kDa protein with multiple isoforms and, in mutant form, has been studied primarily as a genetic risk factor for ovarian and breast cancers^9^. BRCA1 binds directly to DSBs bearing the histone variant γH2A.X, recruiting other repair factors and chromatin modifiers to suppress nuclease activity around DSBs and promote repair^9^,^10^. In proliferating cells, BRCA1 promotes DSB repair by homologous recombination^9^,^11^, an unlikely event in differentiated neurons^2^. In developing brain, BRCA1 is required for neuronal progenitors to survive^12^. Its roles in mature brain are largely unknown. Striatal BRCA1 levels are altered in a Huntington's disease model^10^.

### BRCA1 levels are reduced in the brains of AD patients

**Figure 2:**
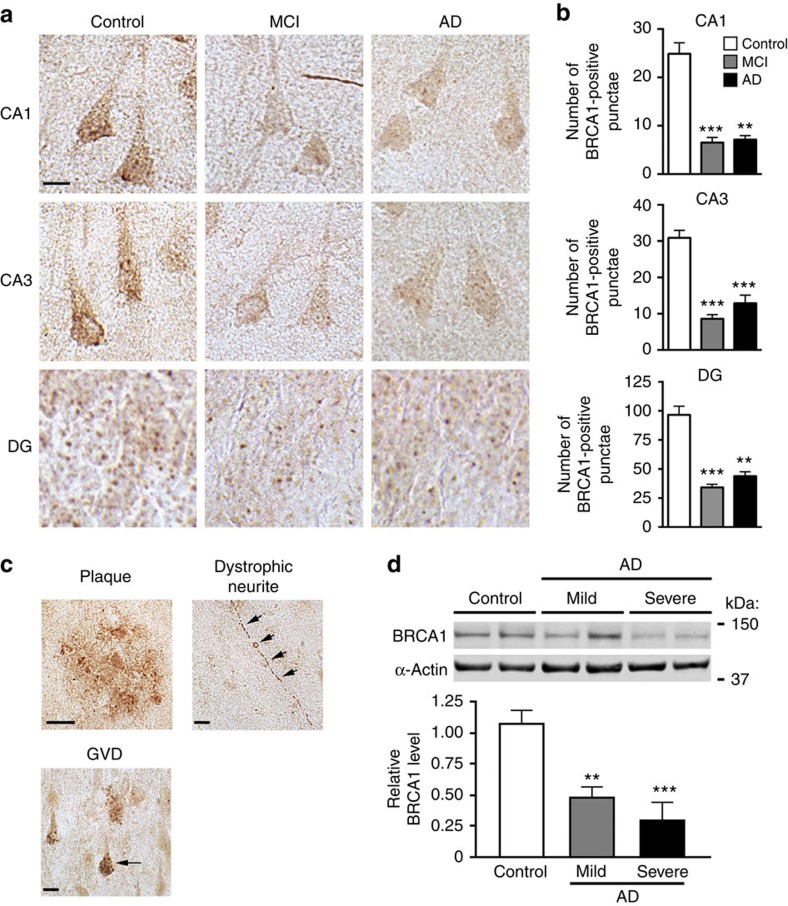
Neuronal BRCA1 reduction in AD patients. (**a–c**) Brain sections containing CA and DG from human cases with CDR/Braak scores of 0/0–I (control), 0.5/0–III (MCI) or +1/IV–VI (AD) were immunostained for BRCA1 (*n*=8 subjects per group). (**a**) Representative micrographs showing BRCA1-immunoreactive neurons in the CA1, CA3 and DG regions of the hippocampus. Scale bar, 10 μm. (**b**) Immunoreactive punctae were counted in 100 cells per subject and region. The average number of punctae per cell (CA1 and CA3) or per 100 cells (DG) is shown. (**c**) Representative micrographs of brain sections from AD patients showing BRCA1 immunoreactivity of an amyloid plaque, a dystrophic neurite (arrowheads), and a neuron with granulovacuolar degenerative (GVD) alterations (arrow). Scale bars, 10 μm. (**d**) BRCA1 levels in the parietal cortex of humans with Braak scores of 0–1 (control, *n*=9), 2–5 (mild to moderate AD, *n*=5) or ≥6 (severe AD, *n*=8) determined by western blot analysis. The average BRCA1 to α-actin ratio in controls was defined as 1.0. **P<0.01, ***P<0.001 versus control by Dunnett test. Bars represent means±s.e.m.

To determine whether BRCA1 is altered in humans with AD, we immunostained post-mortem brain sections from people who had no cognitive deficits and a Braak score of 0 (controls) and from patients with mild cognitive impairment (MCI) or AD with an antibody against BRCA1. Hippocampal neurons showed punctate BRCA1 immunoreactivity in the nucleus and cytoplasm (Fig. 2a and Supplementary Fig. 2a,b). In neuronal cell bodies, the number of BRCA1-immunoreactive punctae in MCI and AD patients was 65% lower in the CA1 and CA3 regions and 75% lower in the DG than in controls (Fig. 2b). In both MCI and AD patients, BRCA1 reductions were comparable in neuronal nuclei and cytoplasm in CA1, but significant only in neuronal nuclei in the DG (Supplementary Fig. 2b,c), probably because nuclei occupy most of the cell body in dentate granule cells.

In contrast to the reduced BRCA1 levels we identified in neurons that still showed grossly normal morphologies, BRCA1 immunoreactivity was increased in several histopathological lesions typically seen in AD brains, such as amyloid plaques, dystrophic neurites and granulovacuolar degeneration (Fig. 2c), consistent with previous reports^13^,^14^. Overall, though, BRCA1 levels in the inferior parietal cortex were 50–70% lower in MCI and AD patients than in controls (Fig. 2d), suggesting that the predominant change in these patients is the neuronal depletion of BRCA1.

### BRCA1 knockdown increases neuronal DSBs

To assess pathophysiological consequences of reduced neuronal BRCA1 levels, we used lentiviral vectors expressing enhanced green fluorescent protein (eGFP) and one of two distinct anti-BRCA1 shRNAs (sh1 or sh2) to knock down BRCA1. In proliferating ovarian carcinoma cells, expression of sh1 had the same effects as genetic ablation of BRCA1 (ref. 15). In primary neuronal cultures, sh1 and sh2 reduced BRCA1 mRNA and protein levels (Supplementary Fig. 3a–c) and BRCA1 immunoreactivity in both the nucleus and the cytoplasm (Supplementary Fig. 3d,e).

**Figure 3:**
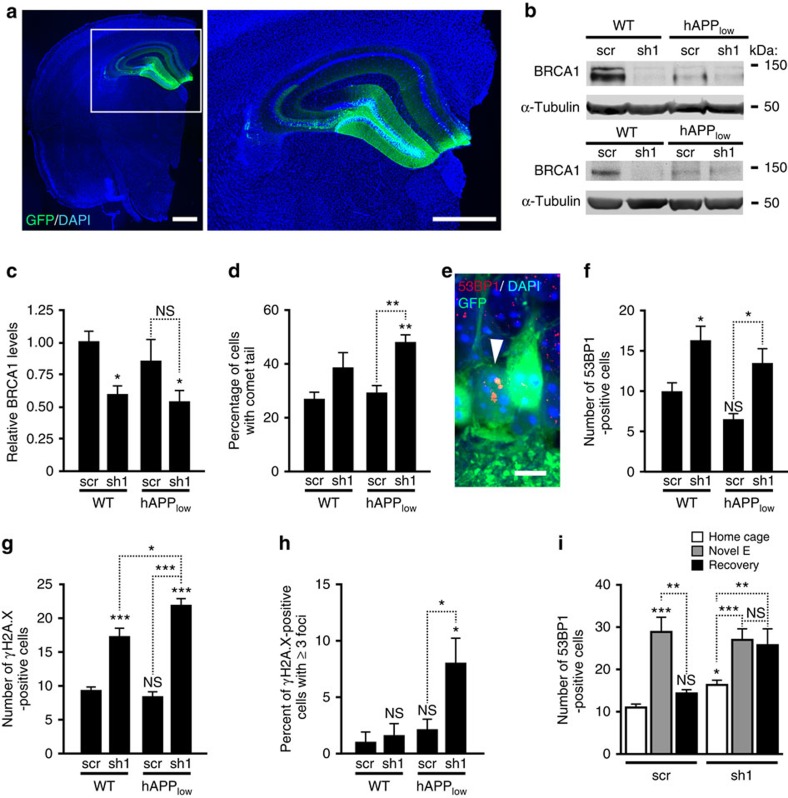
Knockdown of BRCA1 increases neuronal DSBs in the DG of mice. WT and hAPP_low_ mice received stereotaxic injections of lentivirus encoding eGFP plus anti-BRCA1 shRNA (LV-shBRCA1-GFP (sh1)) or scrambled shRNA (LV-Scr-GFP (scr)) into the DG at 1–2 months of age and were analysed 3 months later. (**a**) Sh1 expression (green) in a DAPI-labelled (blue) coronal brain section from a WT mouse was visualized by fluorescence microscopy. Scale bar, 1 mm. (**b**) Western blots illustrating particularly effective knockdowns of BRCA1 in the DG. A unit of 50 (top) or 20 μg (bottom) of total protein was loaded per well. (**c**) BRCA1 levels in the DG were normalized as in Fig. 1b. *n*=14–17 mice per genotype and treatment from three cohorts. (**d**) DSB levels in cell nuclei isolated from DG homogenates were assessed by comet assay at neutral pH. The percent of nuclei with comet tails, reflecting DNA fragmentation, is shown (*n*=3–5 mice per genotype and treatment). (**e**) Confocal micrograph of granule cells in the DG of a WT-sh1 mouse showing three typical 53BP1-immunoreactive foci (red) in the nucleus of one of the neurons (white arrowhead), GFP immunostaining of transduced cells (green) and DAPI labelling of nuclei (blue). Scale bar, 10 μm. (**f**–**i**) Dentate granule cells with 53BP1-positive (**f**,**i**) or γH2A.X-positive (**g**,**h**) foci were counted in three (**f**) or two (**g–i**) sections per mouse. (**f**) Number of granule cells per section with 53BP1-positive foci (*n*=7–10 mice per genotype and treatment). (**g**) Number of granule cells per section with γH2A.X-positive foci (*n*=4–6 mice per genotype and treatment). (**h**) Percentage of γH2A.X-positive cells per section with ≥3 γH2A.X foci (*n*=4–6 mice per genotype and treatment). (**i**) Number of granule cells per section with 53BP1-positive foci in scr- or sh1-injected WT mice analysed after they remained in their home cage, explored a novel environment for 2 h (Novel E), or explored the novel environment for 2 h and were returned to their home cage for 24 h (Recovery). *n*=4–13 mice per condition. *P< 0.05, **P<0.01, ***P<0.001 versus leftmost bar or as indicated by brackets (Bonferroni test). NS, not significant. Bars represent means±s.e.m.

We injected sh1 or sh2 bilaterally into the DG of WT mice (Fig. 3a). To determine whether mice with moderate neuronal overexpression of human hAPP/Aβ have increased sensitivity to neuronal BRCA1 knockdown, we also injected hAPP mice from the lower expresser line J9 (hAPP_low_), which have minimal or no behavioural deficits at baseline^16^. Mice injected with a lentiviral vector expressing eGFP and scrambled shRNA (scr) served as negative controls.

Mice were injected at 1–2 months (scr and sh1 comparison) or 2.5–4 months (scr, sh1 and sh2 comparison) of age and analysed 1–3 months later. As with similar viral vectors encoding other shRNAs that we used in previous studies^6^, 50–80% of granule cells were GFP positive within 1 month after the injection regardless of the shRNA or the group of mice injected. The majority of GFP-positive cells were neurons, as judged from the shapes of their cell bodies and processes, the size of their nuclei and the co-localization of GFP with microtubule-associated protein 2 (MAP2) (Supplementary Figs 3d and 4a), a specific marker of neuronal cell bodies and dendrites.

Two to three months after viral injections, BRCA1 levels in the DG were 40–50% lower in WT mice expressing sh1 or sh2 than in WT mice expressing scr (Fig. 3b,c and Supplementary Figs 4b–e). The BRCA1 signal ratio of transduced to nontransduced neurons was 40% lower in sh1-injected WT and hAPP_low_ mice than in scr-injected WT and hAPP_low_ mice (Supplementary Fig. 4c–e). BRCA1 levels in the DG were lower in uninjected hAPP-J20 mice than in uninjected WT mice (Fig. 1b), but not significantly different in scr-injected hAPP_low_ and WT mice (Fig. 3c and Supplementary Fig. 4b), although hAPP_low_ mice showed a trend towards lower levels. BRCA1 levels were slightly, but insignificantly, lower in sh1-expressing hAPP_low_ mice than in sh1-expressing WT mice (Fig. 3c). Thus, expression of either of two shRNAs targeting BRCA1 mRNA effectively lowered BRCA1 levels, and Aβ-dependent reductions in BRCA1 may require a certain threshold level of Aβ expression.

To determine whether neuronal knockdown of BRCA1 affects DNA integrity, we measured nuclear DNA fragmentation in a comet assay at neutral pH^17^. We isolated cell nuclei from DG and determined the proportion of nuclei with comet tails and the length of the tails (Fig. 3d and Supplementary Fig. 5a,b), which reflect the prevalence and extent of DSBs, respectively^17^. BRCA1 knockdown increased the proportion of nuclei with comet tails in hAPP_low_ mice and was associated with a trend in this direction in WT mice (Fig. 3d). The severity of DNA damage, reflected in the length of comet tails, did not differ significantly among the groups (Supplementary Fig. 5b).

We also immunostained brain sections with antibodies against 53BP1 and γH2A.X, which form nuclear foci around DSBs (Fig. 3e)^4^,^11^. sh1-injected mice had 1.6-fold (WT) to 2-fold (hAPP_low_) more cells with 53BP1-positive foci per DG section than scr-injected controls (Fig. 3f). sh1-injected mice had 2-fold (WT) to 2.6-fold (hAPP_low_) more cells with γH2A.X-positive foci per DG section than scr-injected controls (Fig. 3g). Baseline numbers of cells with γH2A.X-positive foci in scr-injected mice were slightly higher than those we previously observed in uninjected WT mice^4^, possibly due to the viral infection *per se* or the co-expression of eGFP. hAPP_low_ expression increased both the number of cells with foci and the percentage of cells with ≥3 γH2A.X-positive foci in sh1-injected mice (Fig. 3g,h), suggesting that even low levels of hAPP/Aβ overexpression can exacerbate the severity of DNA damage when BRCA1 levels are reduced. Similar results were obtained with sh2 (Supplementary Fig. 5c–e). Taken together, these findings suggest that BRCA1 critically contributes to DSB repair in central neurons.

The increase in DSBs caused by sh1 expression in WT and hAPP_low_ mice was smaller than the threefold increase in DSBs detected by comet assay and 53BP1 immunostaining in uninjected hAPP-J20 mice, as compared with uninjected WT controls^4^. This difference probably reflects a BRCA1 dose effect, as levels of BRCA1 in the DG were lower in uninjected hAPP-J20 mice (Fig. 1a) than in sh1-injected WT and hAPP_low_ mice (Fig. 3c).

Physiological increases in neuronal activity transiently increase neuronal DSBs in WT mice^4^. To test the role of BRCA1 in the repair of DSBs generated in response to physiological activation, we let sh1- or scr-injected WT mice explore a novel environment for 2 h. In all mice the number of dentate granule cells with 53BP1 foci increased immediately after the exploration (Fig. 3i). When replicate groups of mice were analysed 24 h after the exploration, the number of neurons with 53BP1-positive foci had returned to baseline levels in scr-injected mice but not in sh1-injected mice (Fig. 3i), suggesting that BRCA1 contributes to the repair of activity-induced DSBs.

### Knockdown of BRCA1 does not cause neuronal apoptosis

Since physiological increases in neuronal activity transiently increase neuronal DSBs in WT mice, it is unlikely that moderate increases in DSBs cause neuronal loss. Furthermore, hAPP-J20 mice have decreased hippocampal BRCA1 levels (Fig. 1a,b) and an increased proportion of neurons with DSBs^4^, but no overt neuronal loss^7^. Consistent with these observations, we found no increases in TUNEL-stained dentate granule cells in sh1-injected WT and hAPP_low_ mice (Supplementary Fig. 6).

Since neurogenesis in the subgranular zone of the DG continues into adulthood and newly born granule cells appear to be more sensitive to DNA damage than mature granule cells^12^, we immunostained brain sections for doublecortin, a specific marker of newly born neurons^18^. sh1 tended to decrease the number of doublecortin-positive cells in WT and hAPP_low_ mice but not significantly (Supplementary Fig. 7). Immunostaining of brain sections from the same mice revealed significantly more 53BP1-positive neurons and less BRCA1 immunoreactivity in sh1-injected mice (Fig. 3f and Supplementary Fig. 4b,c), confirming the effectiveness of the BRCA1 reduction.

### BRCA1 reduction causes learning and memory deficits in mice

**Figure 4:**
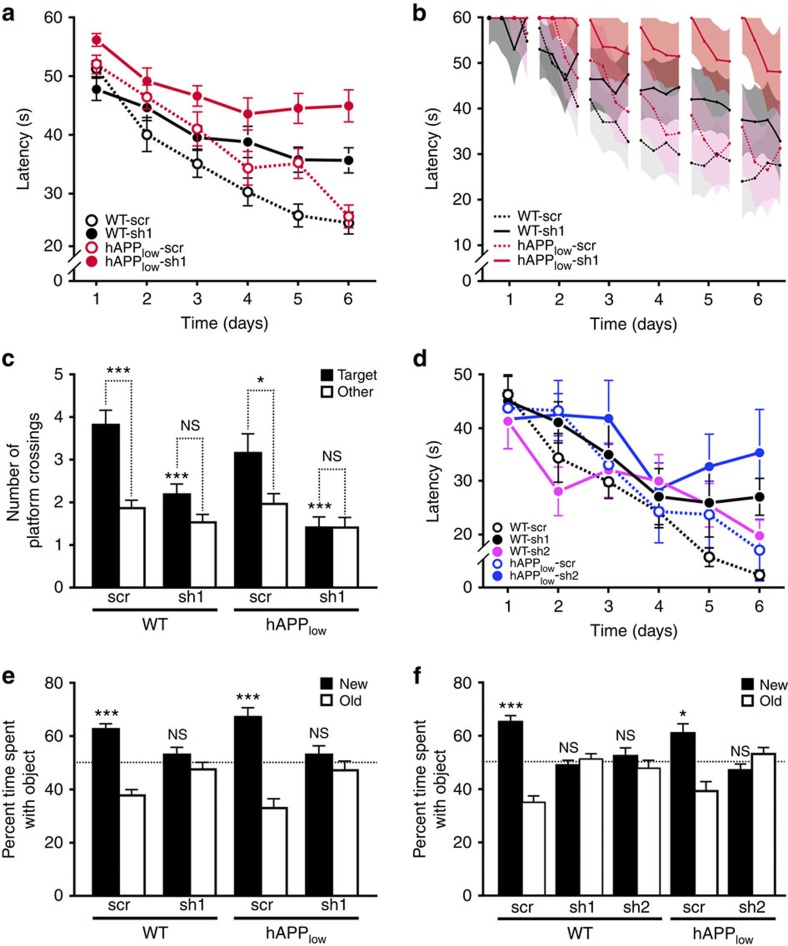
Knockdown of BRCA1 in DG impairs spatial learning and memory in mice. Mice (*n*=18–28 per genotype and treatment) received injections of scr or sh1 into the DG at 1–2 months of age (**a**–**c**,**e**) as in Fig. 3. An independent cohort of mice (*n*=6–11 per genotype and treatment) was treated similarly to compare the effects of scr, sh1 and sh2 (**d**,**f**). Mice were tested in the MWM (**a**–**d**) and a novel place recognition test (**e**,**f**) either 1 (**a**–**d**) or 2 (**e**,**f**) months after injection. (**a**) Learning curves show mean daily escape latencies calculated from four trials per day. (**b**) Trial-by-trial data were analysed with a Bayesian model yielding posterior mean learning curve estimates (lines) and 95% credible intervals (CI, shaded regions). Broken up in segments of four trials per day, learning curve estimates show changes in performance across trials per day and across days. There was no significant interaction between the effects of genotype and treatment on learning rate (*P*=0.16), although sh1 injection tended to affect WT mice less than hAPP_low_ mice (WT: sh1 0.9 s per trial (CI 0.6–1.2) versus scr 1.6 s per trial (CI 1.3–1.9; *P*=0.004); hAPP_low_: sh1 0.4 s per trial (95% CI 0.1–0.7) versus scr 1.6 s per trial (CI 1.2–1.9), *P*<0.0001). (**c**) Number of times mice crossed the original platform (Target) location versus corresponding locations in other quadrants (Other) during a 60-s probe trial 24 h after hidden-platform training. *P<0.05, ***P<0.001 versus same genotype injected with scr (Bonferroni test) or as indicated by brackets (paired *t*-test). (**d**) Learning curves show mean daily escape latencies calculated from four trials per day. (**e**,**f**) Spatial memory assessed in a novel place recognition task 3 h after training. Bars represent the time mice spent exploring the object that was moved (New) or not (Old), expressed as percentage of the total time spent exploring either object. *P<0.05, ***P<0.001 versus chance (dashed line) (one-sample *t*-test). Values are means±s.e.m. (**a**,**c**,**d**–**f**) or means±95% CI (**b**). NS, not significant.

To assess whether reductions in BRCA1 and increases in neuronal DSBs affect cognitive functions, we used the Morris water maze (MWM) and a place recognition paradigm. In the hidden-platform component of the MWM task, which requires both procedural and spatial learning, all groups of mice learned to use extramaze cues to find the platform (Fig. 4a,b). sh1-mediated knockdown of BRCA1 impaired task acquisition in WT and hAPP_low_ mice (Fig. 4a,b; *P*<0.0001 versus scr-injected controls by Bayesian modelling). While the learning curves of scr-injected WT and hAPP_low_ mice were not significantly different (*P*=0.74), sh1-injected hAPP_low_ mice were more impaired than sh1-injected WT mice (*P*<0.05), suggesting a greater susceptibility of hAPP_low_ mice to BRCA1 knockdown. sh1-injected hAPP_low_ mice performed as poorly as uninjected hAPP-J20 mice^6^,^7^, which have higher levels of Aβ in the brain^19^ and reduced BRCA1 levels in the DG at baseline (Fig. 1a).

A more detailed analysis of trial-by-trial data by Bayesian modelling revealed additional differences among the groups (Fig. 4b). On most training days, all groups improved their performance across the four daily trials (*P*<0.0001). However, from one day of training to the next, hAPP_low_ mice had greater setbacks in escape latency than WT mice. While in scr-injected hAPP_low_ mice there was only a trend in this direction (Supplementary Fig. 8a), in sh1-injected hAPP_low_ mice the difference in inter-day setbacks became significant (*P*=0.02) during the last 3 days of training (Supplementary Fig. 8b), suggesting that BRCA1 reduction elicits or exacerbates an hAPP/Aβ-dependent deficit in memory consolidation.

In a probe trial (platform removed) 24 h after the end of training, sh1-injected WT and hAPP_low_ mice showed less preference for the original platform location (Fig. 4c) and the target quadrant (Supplementary Fig. 8c) than scr-injected mice. sh1-injected WT, but not hAPP_low_, mice showed a trend towards crossing the platform location more often than corresponding locations in nontarget quadrants (Fig. 4c). Thus, hAPP_low_ mice may be more susceptible to BRCA1 knockdown.

The four groups did not differ in swim speeds (Supplementary Fig. 8d) or escape latencies to a visibly cued platform (Supplementary Fig. 8e). Nor did they differ in exploration and anxiety-like behaviours in an elevated plus maze, although sh1- and scr-injected hAPP_low_ mice tended to spend more time in the open arms (Supplementary Fig. 8f), an abnormality seen more prominently in hAPP-J20 mice and other high-expresser hAPP lines^16^,^20^.

Knocking down BRCA1 in both DG with a different shRNA (sh2) also caused spatial learning and memory deficits in the MWM (Fig. 4d and Supplementary Fig. 9a–c).

In a novel place recognition task^6^ that engages the DG^21^, mice were allowed to explore two objects within an arena. Three hours later, they were reintroduced into the arena after one of the objects had been moved. Scr-injected WT and hAPP_low_ mice, but not sh1- or sh2-injected mice, spent more time exploring the moved object (Fig. 4e,f)—additional evidence that neuronal BRCA1 reduction in the DG interferes with spatial learning and memory. DG injection with sh1 or sh2 did not significantly alter behaviour in an open field containing no objects (Supplementary Fig. 9d,e).

### Neuronal BRCA1 levels are regulated by neuronal activity

**Figure 5:**
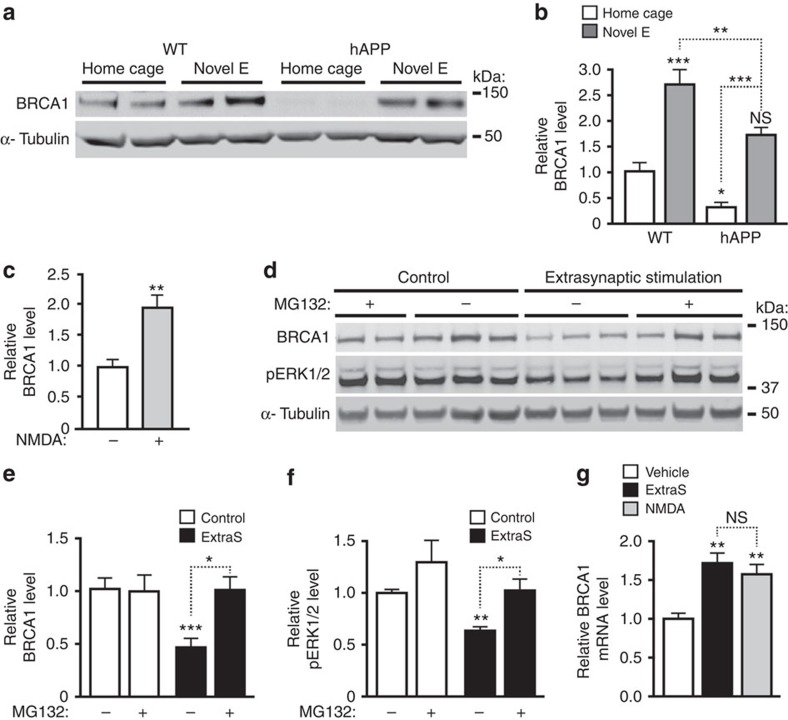
Neuronal activity regulates BRCA1 levels. (**a**,**b**) BRCA1 levels in the DG were determined by western blot analysis in WT and hAPP-J20 mice that remained in their home cage or explored a novel environment for 2 h (Novel E). (**a**) Western blot showing typical activity-induced increases in BRCA1 levels and particularly marked reductions in baseline BRCA1 levels in hAPP mice. (**b**) For quantitation, the average BRCA1 to α-tubulin ratio in WT mice (home cage) was defined as 1.0. *n*=8–19 mice per group from two independent experiments. (**c**) Cultures of primary hippocampal neurons from WT mice were stimulated with NMDA (+) or treated with vehicle (–). One hour later, BRCA1 levels were determined by western blotting. The average ratio of BRCA1 to α-tubulin in vehicle-treated cultures was defined as 1.0. *n*=4–6 wells per condition from two independent experiments. (**d**–**f**) Cultures of primary hippocampal neurons from WT mice were incubated with the proteasome inhibitor MG132 (+) or with vehicle (–) starting 1 h before stimulation of extrasynaptic NMDARs (ExtraS). Four hours later, BRCA1 levels were determined by western blotting. (**d**) Representative western blots. (**e**) Quantitation of BRCA1 protein levels. (**f**) Quantitation of pERK1/2 protein levels to confirm the efficacy of the protocol. The average ratio of BRCA1 or pERK1/2 to α-tubulin in vehicle-treated cultures was defined as 1.0. *n*=4–8 wells per condition from three independent experiments. (**g**) Cultures of primary hippocampal neurons from WT mice were treated as indicated, and BRCA1 mRNA levels were quantitated by RT–qPCR. The average BRCA1 to GAPDH mRNA ratio in vehicle-treated cultures was defined as 1.0. *n*=6 wells per condition from two independent experiments. *P<0.05, **P<0.01, ***P<0.001 versus leftmost bar or as indicated by brackets by Bonferroni test (**b**,**e**,**f**), unpaired *t*-test (**c**) or Dunnett's test (**g**). Bars represent means±s.e.m. NS, not significant.

To assess whether neuronal BRCA1 expression is regulated by neuronal activity, we allowed some WT mice to explore a novel environment for 2 h and kept others in their home cages. Exploratory activity increased BRCA1 levels in the DG (Fig. 5a,b) and N-methyl-D-aspartate (NMDA) treatment increased BRCA1 levels in primary neuronal cultures (Fig. 5c), most likely in response to the neuronal DSB formation caused by this stimulation^4^. Exploration of a novel environment also increased BRCA1 levels in hAPP-J20 mice (Fig. 5a,b), suggesting intact upregulation of BRCA1 in response to DSBs or neuronal activity. However, like baseline levels, BRCA1 levels after exploration were lower in hAPP-J20 than WT mice, which may explain, at least in part, the abnormal persistence of exploration-induced neuronal DSBs in hAPP-J20 mice^4^.

Since our previous findings suggested that abnormal activation of extrasynaptic NMDARs is critically involved in the Aβ-induced accumulation of DSBs^4^, we examined whether activation of these receptors affects BRCA1 levels. Five hours after stimulation of extrasynaptic NMDARs, levels of BRCA1 and pERK1/2 decreased by 50% (Fig. 5d–f). In contrast, BRCA1 mRNA levels were increased 1 h after treatment of cultures with NMDA and 5 h after activation of extrasynaptic NMDARs (Fig. 5g), suggesting a post-transcriptional mechanism of the BRCA1 protein depletion. Because degradation by the ubiquitin proteasome pathway regulates BRCA1 levels in dividing cells^22^ and is also involved in the reduction of pERK1/2 after stimulation of extrasynaptic NMDARs^23^, we pretreated neurons with the proteasome inhibitor MG132. Inhibition of this pathway prevented reductions in both BRCA1 and pERK1/2 (Fig. 5d–f), suggesting that activation of extrasynaptic NMDARs causes proteasomal degradation of BRCA1.

### BRCA1 depletion alters neuronal structure and function

**Figure 6:**
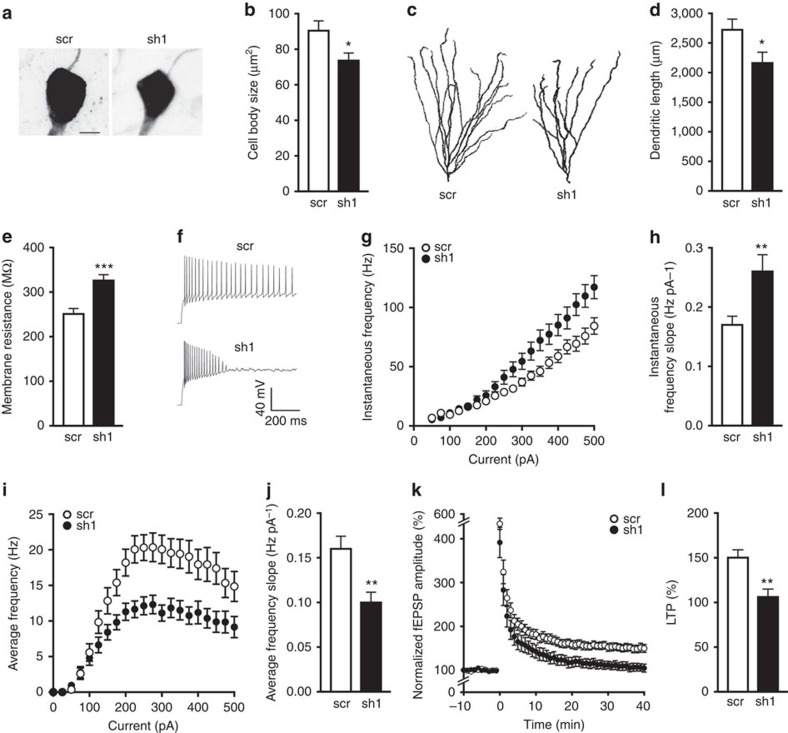
BRCA1 knockdown reduces neuronal size, increases neuronal excitability and impairs LTP. WT mice received injections of scr or sh1 into both DG at 3 months of age as in Fig. 3. Acute brain slices were prepared 45–65 days later. (**a**–**j**) GFP-positive granule cells were targeted for whole-cell patch-clamp recordings, filled with biocytin and immunostained with streptavidin for analysis of cell morphology. (**a**) Representative maximum intensity projection images of cell bodies from streptavidin-labelled granule neurons expressing scr or sh1. Scale bar, 5 μm. (**b**) Quantitation of cell body sizes. *n*=14–16 cells per group (1–6 cells per mouse from 4 mice). (**c**) Representative maximum intensity projection images of traced granule cell dendritic arbours. (**d**) Quantitation of dendritic arbour sizes expressed as the average total length of dendrites per neuron. *n*=10–12 cells per group (1–4 cells per mouse from 4 mice). (**e**–**j**) Whole-cell patch-clamp recordings were used to assess neuronal excitability. Action potential firing was induced with 1.5-s long current stimulation. *n*=30–33 cells per group (3–9 cells per mouse from 5 mice). (**e**) Average membrane resistance of patched granule cells. (**f**) Representative action potential firing of neurons expressing scr or sh1 in response to stimulation with 250 pA. (**g**) Instantaneous frequency (1/interspike interval) as a function of stimulation current. (**h**) Quantitation of the slope of instantaneous frequency versus current curve. (**i**) Average frequency (spikes s^−1^) as a function of stimulation current. (**j**) Quantitation of the slope of average frequency versus current curve. (**k**,**l**) Field potential recordings were performed in the CA3 stratum lucidum while stimulating mossy fibres; LTP was induced with 125 pulses at 25 Hz. *n*=12 slices per group (2–3 slices per mouse from 5 mice). (**k**) fEPSP amplitude over time normalized to the average baseline amplitude (−10 to 0 min). (**l**) Quantitation of LTP (average of normalized fEPSP amplitude at 35–40 min). *P<0.05, **P<0.01, ***P<0.001 versus scr by *t*-test. Bars represent means±s.e.m.

Learning and memory deficits can arise from a variety of pathological changes in neuronal morphology and function, and several of these alterations occur in diseases associated with changes in DNA integrity and genomic stability^2^,^24^,^25^. To examine the effect of BRCA1 reduction on neuronal structure and function, we investigated the morphological and electrophysiological characteristics of scr- and sh1-expressing dentate granule cells. We prepared acute hippocampal slices from scr- or sh1-injected WT mice and targeted GFP-positive cells in the DG for whole-cell patch-clamp recordings while simultaneously filling the recorded cells with biocytin. Morphometric analysis of filled neurons revealed that sh1-expressing neurons had smaller cell bodies (Fig. 6a,b) and dendritic arbours (Fig. 6c,d) than scr-expressing neurons.

Consistent with the shrinkage in cell size and dendritic arbour, BRCA1 reduction also increased the resting membrane resistance (Fig. 6e) and intrinsic excitability (Fig. 6f–h and Supplementary Fig. 10a,b) of dentate granule cells. Compared with scr-expressing neurons, sh1-expressing neurons also had a higher instantaneous frequency of action potentials as a function of stimulation current (Fig. 6g,h), lower (more negative) spike threshold (Supplementary Fig. 10a) and reduced spike after hyperpolarization (Supplementary Fig. 10b). Interestingly, as shown in the representative action potential traces (Fig. 6f), reduction of BRCA1 levels also impaired the cells' ability to sustain prolonged action potential firing, thereby leading to a decrease in the average firing frequency over time (Fig. 6i,j).

To examine circuit modifications that may arise from the observed cellular alterations and may underlie cognitive impairments in sh1-injected mice, we assessed synaptic plasticity between granule cells and CA3 neurons by stimulating mossy fibres and recording field excitatory postsynaptic potentials (fEPSPs) in CA3 stratum lucidum. BRCA1 reduction impaired long-term potentiation (LTP) induced by high-frequency stimulation of mossy fibres (Fig. 6k,l) but did not affect short-term plasticity, paired-pulse facilitation (Supplementary Fig. 10c) or post-tetanic potentiation (Supplementary Fig. 10d). These findings suggest that BRCA1 plays a critical role in learning and memory by maintaining neuronal morphology, firing properties and synaptic plasticity to ensure proper functioning of cells and neural circuits.

### BRCA1 reduction causes abnormal chromatin remodelling

Depletion of BRCA1 in the DG of hAPP-J20 mice was associated with markedly increased dimethylation of Lys9 in histone 3 (me2H3(K9)) (Fig. 1a,b). DSB formation triggers repression of gene expression by dimethylations of histone 3 (ref. 11). Because these modifications last until DSB repair is complete^11^, the increased levels of me2H3(K9) in hAPP-J20 mice likely reflect diminished repair of DSBs and prolonged chromatin remodelling.

**Figure 7:**
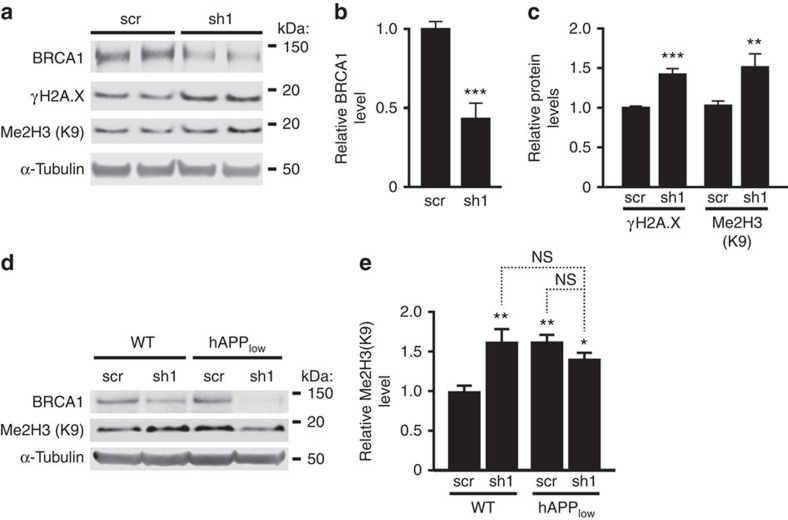
BRCA1 knockdown causes abnormal chromatin remodelling *in vitro* and *in vivo*. (**a–c**) Cultures of primary hippocampal neurons from WT mice were infected with sh1 or scr on DIV 7 and analysed 7 days later. Multiplicity of infection: 1 TU per cell. (**a**) Representative western blots. (**b**) Quantitation of BRCA1 protein levels. *n*=15–17 wells per condition from 7 independent experiments. (**c**) Quantitation of γH2A.X and Me2H3(K9) levels. *n*=9–17 wells per condition from 7 independent experiments. (**d**,**e**) WT and hAPP_low_ mice received bilateral injections of scr or sh1 into the DG at 1–2 months of age and were analysed by western blotting 3 months later. (**d**) Representative western blots. (**e**) Quantitation of Me2H3(K9) levels. *n*=14–17 mice per genotype and treatment from three cohorts. The average ratio of BRCA1 or histone to α-tubulin in scr-infected cultures (**b**,**c**) or scr-injected WT mice (**e**) was defined as 1.0. *P<0.05, **P<0.01, ***P<0.001 versus corresponding scr condition bar by unpaired *t*-test (**b**,**c**) or versus leftmost bar by Bonferroni test (**e**). Bars represent means±s.e.m. NS, not significant.

To test this hypothesis, we reduced BRCA1 levels in primary cultures of hippocampal neurons by transduction with sh1, using scr as a negative control (Fig. 7a–c and Supplementary Fig. 3a–c). The sh1-induced BRCA1 reduction (Fig. 7a,b) was similar to that caused by Aβ (Fig. 1d) and increased γH2A.X and me2H3(K9) levels (Fig. 4c). Similar increases in me2H3(K9) were detected in the DG of sh1-injected WT mice and of sh1- or scr-injected hAPP_low_ mice (Fig. 7d,e). Total histone 3 levels in the DG were similar across genotypes and treatments (Supplementary Fig. 11a,b). Thus, BRCA1 reduction can elicit histone modifications known to cause gene repression^26^, and this effect is exacerbated in the context of pathologically elevated levels of Aβ.

To further explore the relationship between me2H3(K9) formation and DSBs, we measured me2H3(K9) and γH2A.X immunoreactivities in dentate granule cells after mice received 0, 1 or 5 Gy of full-body γ-irradiation. The number of γH2A.X foci increased with the dose of radiation, whereas the number of me2H3(K9) immunoreactive punctae reached maximal levels at 1 Gy and did not increase further at 5 Gy (Supplementary Fig. 11c,d). These results confirm in neurons that me2H3(K9) is related to the formation of DSBs^27^.

## Discussion

Our study shows that BRCA1 critically contributes to DSB repair in central neurons and that neuronal reductions in BRCA1 cause increased persistence of DSBs, abnormal chromatin remodelling, cellular dysfunction and cognitive deficits. It also identifies depletions of BRCA1 in brains of patients with MCI or AD, and provides evidence that these depletions are caused by the pathological accumulation of Aβ, which may promote the proteasomal degradation of BRCA1 through overactivation of extrasynaptic NMDA receptors.

In our experimental models, we reduced BRCA1 expression specifically in the DG for several reasons. Although dentate granule neurons are relatively resistant to cell death in AD, there is plenty of evidence for their dysfunction in this condition. For example, functional magnetic resonance imaging studies showed profound alterations in the DG of patients with MCI or early AD^28^,^29^,^30^. Furthermore, patients with AD have several molecular abnormalities in dentate granule cells that are also found in hAPP-J20 mice, including depletion of BRCA1 (this study), reduction of calbindin and increased expression of collagen VI, metenkephalin and group IV phospholipase A2 (refs 5, 7). Degeneration of the perforant path, the main afferent input to the DG, is a well-established feature of AD^31^,^32^. The DG also shows the greatest increase in neuronal DSBs in hAPP mice^4^, and can readily be transduced with lentiviral vectors^6^,^33^. In addition, several sensitive behavioural assays exist to evaluate its function and dysfunction^21^,^34^.

Interestingly, knockdown of BRCA1 in both DG caused deficits in spatial learning and memory in WT mice. In exploring the mechanisms that may underlie these abnormalities, we discovered that the size of cell bodies and dendritic arbours was reduced in neurons expressing low levels of BRCA1. Changes in dendritic complexity, including length and branching, also occur in AD patients^35^, AD-related animal models^36^ and other neurodegenerative diseases^37^,^38^.

The reductions in the size of cell bodies and dendritic arbours were associated with an increase in cell excitability, consistent with results from computational modelling^39^,^40^ and experimental studies^40^. Furthermore, neuronal BRCA1 reduction impaired long-term synaptic plasticity, which is involved in hippocampus-dependent associative spatial learning and memory^41^,^42^. Additional studies are needed to determine whether these neuronal alterations result from faulty DSB repair or from reductions in other functions BRCA1 fulfils in neurons. Notwithstanding this uncertainty, the alterations in synaptic plasticity and neuronal excitability we observed after BRCA1 reduction may well contribute to the circuit and network dysfunctions that underlie cognitive deficits and behavioural alterations in AD and related animal models^5^,^30^,^43^,^44^.

The fact that select manipulations in the DG caused behavioural deficits in the MWM may seem at odds with reports that complete removal or destruction of both DG does not prevent mice from solving this task^45^. However, diverse lines of evidence suggest that solving this task actively engages the DG^46^,^47^. Other molecular manipulations of both DG also altered performance in this or other tasks requiring the encoding of spatial contexts^6^,^34^. Conceivably, compensatory mechanisms in the remainder of the brain are engaged more effectively when a brain region is eliminated than when it is dysfunctional but still connected to the network. Indeed, individual neurons can affect global network activity^34^.

BRCA1 is critical for DNA repair in dividing cells, in which BRCA1 deficiency leads to cell death^9^,^11^. If it had a similar role in mature neurons, Aβ- and sh1-induced BRCA1 reductions might impair cognitive functions through neuronal apoptosis resulting from accumulating DNA damage. However, despite having impaired learning and memory, WT and hAPP_low_ mice with reduced BRCA1 levels showed no evidence for neuronal apoptosis or loss. Interestingly, BRCA1 reduction impaired the repair of neuronal DSBs elicited by physiological brain activity in WT mice. It is tempting to speculate that much of the neuronal DNA damage that accumulates in brains with reduced BRCA1 levels represents these circumscribed activity-induced DSBs^4^ and that chronic accumulation of such DSBs could ultimately contribute to neuronal degeneration, for example, by dysregulating gene expression^24^.

BRCA1 knockdown tended to reduce the number of newborn granule cells in the DG, but this trend was not statistically significant. In addition, sh1-injected WT mice and scr-injected hAPP_low_ mice had the same level of doublecortin-positive neurons in the DG (Supplementary Fig. 7), but only the former group had significant deficits in the MWM probe trial and novel place recognition task (Fig. 4), suggesting a dissociation between neurogenesis and sh1-induced behavioural deficits. Furthermore, even profound suppression of neurogenesis appears to cause only limited cognitive impairments^48^. It therefore seems most likely that the effects of BRCA1 knockdown on learning and memory reflect changes in neuronal structure and function rather than neuronal loss and impaired neurogenesis.

It is tempting to speculate that BRCA1-dependent alterations in neuronal structure and function involve chromatin remodelling and changes in gene expression. Indeed, the functions and roles of neuronal activity-associated formation and repair of DSBs may relate to chromatin remodelling, coordinated transcription or silencing of gene clusters, or movement of transposable elements^24^,^49^,^50^. By preventing effective repair of activity-dependent DSBs, neuronal depletion of BRCA1 may dysregulate these dynamic processes.

In many non-neuronal cell types, DSB formation is associated with increased dimethylation on lysine 9 (K9) of histone 3 (ref. 27). We found that this association also exists in mature post-mitotic neurons. In dividing cells, me2H3(K9) formation is critically involved in BRCA1-dependent repair processes^26^ and mediates gene repression^51^. Although transient me2H3(K9) formation in neurons is linked to memory acquisition^52^, abnormal persistence of this modification in neurons has been implicated in behavioural and neuropsychiatric disorders. In our study, BRCA1 reduction increased neuronal me2H3(K9) levels *in vitro* and *in vivo*, possibly because of the increased persistence and accumulation of neuronal DSBs. However, me2H3(K9) levels in the DG were higher in scr-injected hAPP_low_ mice than scr-injected WT mice, even though these groups had comparable numbers of neurons with DSBs. Conceivably, it may take less Aβ elevation and BRCA1 reduction to cause dimethylation of lysine 9 on core histone 3 than to promote the accumulation of DSBs. Alternatively, the detection of me2H3(K9) by western blot analysis may simply be more sensitive than γH2A.X immunohistochemistry or the comet assay. It is also worth noting in this context that irradiated WT mice had many more me2H3(K9) punctae in the DG than γH2A.X-positive foci. This dissociation could also reflect differences in the sensitivity of the methods used to detect the alterations. It may also suggest that, although me2H3(K9) is clearly related to the formation of DSBs, it also forms part of additional processes that are engaged by pathologically elevated Aβ levels and γ-irradiation.

The finding that BRCA1 critically contributes to DSB repair in central neurons was unexpected, as BRCA1 is presumed to be involved primarily in DNA repair by homologous recombination, which is not known to occur physiologically in non-dividing cells; in mature neurons, DSBs appear to be repaired by non-homologous end joining (NHEJ)^2^. However, to our knowledge, the role of BRCA1 in NHEJ has been studied only in mitotic cells, where it is important for the fidelity of the canonical NHEJ repair pathways^11^,^53^,^54^,^55^. BRCA1 also participates in non-classical NHEJ pathway: microhomology-mediated end joining and alternative NHEJ. These pathways allow for genomic stability-conserving repair and require the functions of BRCA1 and 53BP1 (refs 56, 57, 58). Additional studies are needed to determine the exact pathway(s) through which BRCA1 contributes to the repair of DSBs in mature post-mitotic neurons.

Another intriguing question our study raises is whether mutations in BRCA1 that increase the risk for certain cancers by impairing DNA repair in highly proliferating cells^9^ also change the function of BRCA1 in the brain. However, unlike the AD-associated depletion of BRCA1 in ageing brains and the experimental knockdown strategy used here, BRCA1 mutations are present from early stages of embryonic development and thus, might allow for the compensatory implementation or activation of alternative DNA repair factors and mechanisms, at least in neurons. Finally, BRCA1 is a complex protein that may have functions in the nervous system distinct from those it has in DNA repair and chromatin remodelling. Additional studies are needed to address these possibilities and explore the therapeutic potential of enhancing DNA repair in AD and related conditions.

## Methods

### Human post-mortem tissues

For BRCA1 immunostaining, sections of 4% paraformaldehyde-fixed post-mortem brain tissues containing hippocampus from people without cognitive impairments (controls, *n*=8, 82.1±5.6 years, mean±s.e.m) and from patients with MCI (*n*=8, 87.6±1.5 years) or AD (*n*=8, 82.0±2.6 years) were obtained from the Alzheimer's Disease Research Center of the University of California at San Diego. Clinical diagnoses were based on the criteria described by Albert *et al*.^59^ and McKhann *et al*.^60^, and neuropathological diagnoses were based on the criteria described by Hyman *et al*.^61^. Cases met the following criteria: controls, clinical dementia rating (CDR) 0 and Braak stage 0–I; MCI cases, CDR 0.5 and Braak stages I–III; and AD cases, CDR ≥1 and Braak stages IV–VI.

For BRCA1 western blot analysis, inferior parietal cortex was dissected from frozen post-mortem brain tissues of non-demented controls (*n*=9, age 81.1±4.4 years) and patients with mild AD (*n*=5, age 84.3±2.2 years) or severe AD (*n*=8, age 71.7±6.1 years) obtained from the Alzheimer's Disease Research Centers of the University of California at San Diego and the New York Brain Bank of Columbia University, New York. AD was diagnosed and its severity scored by Braak staging based on the extent of amyloid plaques and neurofibrillary tangles in formalin-fixed tissue from the opposite hemibrain according to criteria of the Consortium to Establish a Registry for Alzheimer's Disease and the National Institute on Aging. Cases met the criteria described above.

### Mice and brain tissues

We studied 4- to 8-month-old heterozygous hAPP transgenic mice and WT mice from lines J9 (hAPP_low_) and J20 (refs 7, 16, 19) on a C57BL6/J background. For γ-irradiation, we used 4.5- to 5.5-month-old WT mice from line J20 (ref. 4). For electrophysiological recordings, we used acute hippocampal slices from 3- to 5-month-old WT mice from line J9. *In vivo* analyses were carried out on sex-matched or sex-balanced groups. Littermates were group housed. For testing in the MWM, mice were single-housed from 5 days before the start of training until the test was concluded. All mice had *ad libitum* access to food (PicoLab Rodent Diet 20, 5053) and water and were exposed to a 12-h light/dark cycle. For histological and biochemical analyses, mice were anesthetized with Avertin (tribromoethanol, 250 mg kg^−1^) and perfused transcardially with 0.9% NaCl. One hemibrain was used fresh for the comet assay or snap frozen and stored at –80 °C for western blot or quantitative PCR with reverse transcription (RT–qPCR) analysis. For RT–qPCR analysis, mice were perfused with 0.9% NaCl containing 0.1% diethylpyrocarbonate. The other hemibrain was drop fixed in 4% paraformaldehyde in PBS and sectioned (30 μm) with a sliding microtome (Leica SM2000R). All mouse experiments were approved by the Animal Care and Use Committee of the University of California, San Francisco.

### Lentiviral constructs and stereotaxic injections

Six anti-*Brca1* shRNAs were obtained from Sigma-Aldrich (Mission shRNA lentiviral particles, parental vector on pLKO.1-purobackbone). Their ability to reduce BRCA1 protein levels was assessed in primary mouse neuronal cultures 1 week after infection. The sequences of the two most effective shRNAs (sh1 and sh2) were: sh1: 5′-CCACAGGTAAATCAGGAATTT-3′ (ref. 15) and sh2: 5′-GTGCTTCCACACCCTACTTAC-3′. The sh1 sequence is in the coding sequence of BRCA1 mRNA, and the sh2 sequence is in the 3′-untranslated region. The sequence of the control, scrambled shRNA was 5′-CCACTACCGTTGTTATAGGTG-3′. Each sequence was inserted into the FUGW plasmid backbone and used to generate lentiviral vectors as described^6^,^62^. Lentiviruses were bilaterally injected stereotaxically into the DG as described^6^, using 1.5 × 10^6^ titre unit (TU) per DG. No specific method of randomization was used to determine the treatment of each mouse. GFP expression was used as an indication of viral transduction. Note, however, that the GFP intensity does not reliably reflect the abundance of shRNA expressed in transduced cells, as the promoters driving the expression of these transgenes are activated by different RNA polymerases: GFP expression is RNA polymerase II dependent, whereas expression of the shRNA is RNA polymerase III dependent.

### Behavioural testing

For all behavioural tests and subsequent analyses, experimenters were blinded to the genotype and treatment of mice. Mice were assessed in the following tests in the indicated sequence and as described in the corresponding references: elevated plus maze^6^, open field^6^, MWM^6^, novel place recognition^6^ and exploration of a novel environment^4^, with the following modifications. In the MWM, hidden-platform training was extended to 6 days (from 5 days) to allow WT mice to reach a steady plateau in their performances without overtraining. The cued-platform training (two trials per day) was done during the 2 days following the probe trial, which was carried out 24 h after the hidden-platform training. Exploration of a novel environment was done 3 weeks after the novel place recognition test. Two cohorts of WT and transgenic mice from line J9 were assessed in all tests in the sequence indicated above. An additional cohort from this line underwent all tests except for the elevated plus maze. To compare the effects of the two shRNAs, an additional cohort from this line underwent all tests except for the elevated plus maze, which was replaced by open field testing. Two behaviourally naive cohorts of mice from line J9 were used for biochemical analyses only. Mice from line J20 were tested only in the novel environment paradigm.

### Cell cultures and treatments

Primary cultures of hippocampal neurons were established as described^4^,^6^, using postnatal day 0 pups from pure C57BL6/J or C57BL6 × FVB/NJ F1 WT mice. Neurons were plated on 12-well plates (Corning) at 0.5 × 10^6^ cells per well or on 12-mm glass coverslips coated with poly-D lysine (Millipore) and laminin (Invitrogen) at low density (150,000 cells per coverslip). Neurons were infected at 7 days *in vitro* (DIV) with lentiviral vectors: 1 TU per cell was used for biochemical analyses and 0.75 TU per cell was used for cell imaging by fluorescence microscopy. Cultures were used for experiments at 14 DIV, 7 days after infection. Experimenters were blinded to treatments. Recombinant Aβ1–42 oligomers were prepared from lyophilized monomers (rPeptide) as described^4^,^6^. NMDA (Sigma-Aldrich; 10 μM final concentration) was applied to neurons for 5 min as described^4^. Some neuronal cultures were treated with the proteasome inhibitor MG132 (Sigma-Aldrich; final concentration 10 μM) starting 1 h before and continuing for 4 h during stimulation of extrasynaptic NMDARs as described^4^ with the following modification. Cultures were not pretreated overnight with tetrodotoxin (TTX), (2R)-amino-5-phosphonovalerate (APV), 2,3-dihydroxy-6-nitro-7-sulfamoyl-benzo[f]quinoxaline-2,3-dione (NBQX), because this pretreatment requires extensive subsequent rinses of cells to wash off the antagonists and leaving out the pretreatment led to more reproducible activation of extrasynaptic NMDARs.

### Western blot analysis

Frozen hemibrains from perfused mice were microdissected in ice-cold PBS containing complete protease inhibitors (Roche) and homogenized in RIPA buffer (10 mM Tris-HCl (pH7.5), 50 mM NaCl, 30 mM Na_4_P_2_O_7_, 5 μM ZnSO_4_, 10% glycerol, 0.1% SDS, 1% Triton X-100 and 1 mM dithiothreitol) containing complete protease inhibitors and a cocktail of phosphatase inhibitors (Sigma-Aldrich). Cultured cells were washed in PBS and scraped into RIPA buffer. Lysates were sonicated for 5 min at 4 °C and spun in a refrigerated microcentrifuge at maximal speed for 10 min. Protein concentrations were determined by Bradford assay.

Proteins (20 μg per mouse sample and 100 μg per human sample) were loaded on 4–12% Bis-Tris gels (Invitrogen), separated by SDS–PAGE, and transferred to nitrocellulose membranes. After 1 h in blocking solution (5% non-fat milk in Tris-buffered saline (TBS)), membranes were incubated with primary antibodies in 5% non-fat milk in TBS/0.1% Tween (or as indicated below) for 3 h at room temperature for monoclonal anti-α-tubulin and anti-γH2A.X antibodies and for polyclonal rabbit anti-Me2H3(K9) (Millipore), or overnight at 4 °C for the other antibodies. Primary antibodies were monoclonal mouse, anti-γH2A.X (JBW301, Millipore; 1:1,000), anti-MRE11 (12D7, Abcam; 1:1,000) and anti-α-tubulin (B5-1-2, Sigma-Aldrich; 1:50,000); polyclonal rabbit anti-α-actin (Sigma-Aldrich; 1:1,000), anti-BRCA1 (Abcam; 1:2,000), anti-γH2A.X (PhosphoDetect, Millipore; 1:1,000), anti-total core histone H3 (Millipore; 1:1,000), anti-phospho P42/44 MAPK (p–ERK1/2; 1:1,000), and anti-NBS1 and anti-RAD51 (Abcam; 1:1,000). Blots were washed three times in TBS/0.1% Tween and incubated with secondary IRD-tagged antibodies (800 IRDye and 680 IRDye) (Li-COR) diluted 1:10,000 in Odyssey blocking buffer for 1 h at room temperature.

Western blot signals were analysed with an Odyssey Li-COR laser-scanning and imaging system (Li-COR, v3.0), except for BRCA1, which could not be detected with the Li-COR system. BRCA1 was detected with secondary horseradish peroxidase-tagged antibodies (Calbiochem). Bands were visualized with an ECL kit (Amersham) and quantified with Image J software. The 23-exon *BRCA1* gene gives rise to different protein isoforms through alternative splicing, and additional products are derived from some of the isoforms by proteolytic cleavage^63^. To quantitate BRCA1 levels, we used the 150-kDa band, which corresponds to the full-length form of the protein. For display in figures, western blots were cropped to focus attention on signals of interest; larger sections of the same blots are shown in Supplementary Figs 12–14.

### RT–qPCR analysis

Total RNA was isolated from microdissected frozen mouse brain tissue (DG or parietal cortex) or from wells of 12-well plates containing primary neurons with the RNeasy Mini kit (Qiagen) and reverse transcribed with random hexamers and oligo(dT) primers. Levels of BRCA1 and GAPDH mRNA were determined with SYBR green PCR reagents and an ABI Prism 7900HT sequence detector according to the manufacturer's instructions (Applied Biosystems). The primer sequences used were BRCA1 forward: 5′-TGCTCATGCCAGCTCATTAC-3′; BRCA1 reverse: 5′-ACTGCTATGCCAGGCTGTTT-3′; GAPDH forward: 5′-GGGAAGCCCATCACCATCTT-3′; and GAPDH reverse: 5′-GCCTTCTCCATGGTGGTGAA-3′.

For quantification, BRCA1/GAPDH ratios were normalized to the average ratio in WT mice or in scr- or vehicle-treated neuronal cultures. The quality of all primers and amplification reactions was assessed by analysis of dissociation curves, standard curve slopes and reactions lacking reverse transcriptase.

### Single-cell gel electrophoresis (comet assay)

The comet assay was carried out at neutral pH as described^4^,^17^ and according to the manufacturer's instructions (Trevigen). At least 150 nuclei per field were counted and scored in 2–3 fields per mouse. The number of nuclei with comets was counted manually and expressed as a percentage of the total number of nuclei inspected in any given mouse. Each comet detected was framed and a cursor was placed on the head of the comet with the selection tool of the Cometscore software (Autocomet). The software automatically determined the tail length using a built-in algorithm for comet scoring. For each field, the experimenter calculated the average tail length.

### Immunocytochemistry

Primary neurons were grown on coverslips for 14 DIV, rinsed with PBS, fixed in 100% methanol for 10 min and rinsed in TBS. Coverslips were incubated in blocking solution (5% normal goat serum in 0.1% TBS-Tween) for 1 h at room temperature and then overnight at 4 °C with rabbit anti-BRCA1 (ABCam), mouse anti-MAP2 (MAB3418, Millipore) and chicken anti-GFP (GFP-1010, Aves Labs) antibodies diluted 1:500 in blocking solution. After rinses with 0.1% TBS-Tween, cells were incubated for 1 h at room temperature with goat anti-mouse Alexa 647-, anti-chicken Alexa 488- and anti-rabbit Alexa 594-conjugated secondary antibodies (1:300, Invitrogen) diluted in 4% normal goat serum in TBS.

Human brain sections were immunostained after antigen retrieval in citrate buffer (pH 6) at 100–115 °C for 15 min and peroxidase inhibition to enhance nuclear staining. Nonspecific binding was blocked by incubation for 2 h in blocking solution (5% normal goat serum in 0.5% TBS-Triton X-100). Sections were incubated overnight at 4 °C with rabbit anti-BRCA1 (Abcam; 1:500 in blocking buffer). Biotinylated anti-rabbit secondary antibody (Jackson ImmunoResearch) was diluted similarly and applied to the sections for 1 h at room temperature. Antibody labelling was visualized with the Avidin–biotin complex kit (Vector Laboratories) and 3,3′-diaminobenzidine tetrahydrochloride (Vector Laboratories).

Immunostaining of mouse brain sections was performed essentially as described^62^. However, PBS was replaced with TBS and steps were added for antigen retrieval and peroxidase inhibition to enhance nuclear staining. Blocking solution was the same as for neuronal cultures except for doublecortin staining, for which the blocking solution was 5% normal donkey serum in 0.25% PBS-Triton X-100. Sections were incubated overnight at 4 °C with primary antibodies, including monoclonal mouse anti-GFP (MAB3580 Millipore), anti-γH2A.X (JBW301, Millipore), anti-MAP2 (MAB3418 Millipore) and anti-NeuN (MAB377, Millipore); polyclonal rabbit anti-53BP1 (Novus Biological), anti-BRCA1 (Abcam) and anti-dimethylated histone H3 (Lys9) (Cell Signaling Technology); and polyclonal goat anti-doublecortin (Santa Cruz Biotechnology). Primary antibodies were diluted 1:500 (or 1:1,000 for anti-doublecortin) in blocking solution. Secondary antibodies for detection of GFP, γH2A.X, MAP2, NeuN, 53BP1, Me2H3(K9) and BRCA1 were goat anti-mouse Alexa 488 or anti-rabbit Alexa 594 (Invitrogen). Biotinylated anti-goat antibodies (Vector Laboratories) were used for doublecortin. Diaminobenzidine (Vector Laboratories DAB kit) was used as a chromogen. After extensive rinses, coverslips or sections were mounted with Vectashield mounting medium with 4,6-diamidino-2-phenylindole (DAPI; Vector Laboratories).

Digitized images of BRCA1 signals were obtained with an all-in-one fluorescence microscopy system (BZ-9000, Keyence) with built-in camera and analysis software (BZII viewer and analyser). Images were acquired with identical exposure settings for BRCA1 signals across all samples in any given experiment. Nuclear and cytoplasmic levels of BRCA1 were quantified, respectively, with the round and freehand selection shape and measurement tools from Image J. The 8-bit image of the red channel, which corresponded to BRCA1 staining, was used to measure BRCA1 levels; the composite image formed from DAPI and GFP or DAPI and MAP2 signals was used to determine whether the optical density (OD) of BRCA1 was measured in a GFP-positive or GFP-negative neuron in the DG or in cell culture. In areas without nuclei in the molecular layer of the DG, background OD was measured with the shape tool described above and subtracted from all BRCA1 OD signals to calculate BRCA1 immunoreactivities in brain sections. In a coverslip stained in the absence of primary antibody, background OD was measured with the shape tools described above, extrapolated to the corresponding areas used for BRCA1 nuclear and cytoplasmic measurements, and subtracted from all BRCA1 OD signals to obtain BRCA1 immunoreactivities in cell cultures.

For quantification of human BRCA1 signals, digitized images of BRCA1 signals were obtained with a digital brightfield Olympus BX41 microscope with a DP11 digital camera system. Counterstaining with haematoxylin was used to differentiate between nucleus and cytoplasm. Nuclear and cytoplasmic regions of neurons on a threshold-adjusted image were manually annotated with the selection shape and measurement tools from Image J. BRCA1-positive punctae in each region were counted semiautomatically with a macro available in Image J. Background signals were measured on sections stained in the absence of primary antibody and used to determine the image threshold.

Super resolution z-stacks of images of 53BP1 foci (6.219 μm, 1,800 × 1,800 pixels), γH2A.X foci and Me2H3(K9) punctae (6.102 μm, 1,240 × 1,240 pixels), and MAP2/GFP co-localization (16.462 μm, 1,552 × 1,552 pixels) were obtained with a Zeiss LSM880 with an Airyscan detector confocal microscope, a × 63 objective, and, for acquisition of 53BP1 or γH2A.X/Me2H3(K9) nuclear signals, an additional × 2.5 zoom. Images were acquired and processed with Zen 2 software. Z-stacks of confocal images were processed with Airyscan, and final images were obtained by orthogonal projection.

53BP1, γH2A.X and doublecortin staining in the DG from coronal sections of mice was quantified essentially as described^4^. Counts of 53BP1- or γH2A.X-positive neurons were averaged from two sections per mouse unless stated otherwise. Counts of doublecortin-positive neurons were averaged from seven sections per mouse.

### TUNEL staining

TUNEL staining was done with the Apoptag Red In Situ Apoptosis Detection Kit (EMD Millipore) according to the manufacturer's instructions. Briefly, floating brain sections were mounted on Superfrost Plus glass slides (Fisherbrand). For the TUNEL reaction, sections were incubated in equilibration buffer before incubation with the TdT enzyme for 1 h at 37 °C in reaction buffer. The reaction was stopped, and the slides were incubated with anti-digoxigenin conjugate (rhodamine) for 30 min at room temperature, washed three times in PBS, blocked with 3% donkey serum for 1 h at room temperature and immunostained overnight with anti-GFP or anti-MAP2 antibodies as described above. Stacks of confocal images were acquired with an MC04 Zeiss LSM510 multiphoton microscope and Zen software. The appearance of DAPI and GFP/MAP2 labels was optimized with z-maximum-intensity projection function of ImageJ.

### Acute slice electrophysiology

*Hippocampal slice preparation*. To ensure optimal health of slices, we prepared acute transverse mouse brain slices (400 μm) with a modified neuroprotective slicing and recovery method as described^64^. Mice were deeply anesthetized with isoflurane and transcardially perfused with 30 ml of chilled oxygenated (95% O_2_, 5% CO_2_) slicing artificial cerebrospinal fluid (ACSF; 92 mM *N*-methyl-D-glucamine, 2.5 mM KCl, 1.2 mM NaH_2_PO_4_, 30 mM NaHCO_3_, 20 mM HEPES, 25 mM glucose, 2 mM thiourea, 5 mM sodium ascorbate, 3 mM sodium pyruvate, 12 mM *N*-acetyl-L-cysteine, 0.5 mM CaCl_2_ and 10 mM MgSO_4_). Brains were quickly removed and sliced with a vibrating microtome (HM650V, Thermo Scientific). Slices were incubated first in slicing ACSF for 10 min at 35 °C and then in recovery ACSF (92 mM NaCl, 2.5 mM KCl, 1.2 mM NaH_2_PO_4_, 30 mM NaHCO_3_, 20 mM HEPES, 25 mM glucose, 2 mM thiourea, 5 mM sodium ascorbate, 3 mM sodium pyruvate, 12 mM *N*-acetyl-L-cysteine, 2 mM CaCl_2_ and 2 mM MgSO_4_) for 1 h at room temperature. Slices were then transferred to a holding chamber containing room-temperature oxygenated recording ACSF (126 mM NaCl, 2.5 mM KCl, 1.25 mM NaH_2_PO_4_, 26 mM NaHCO_3_, 12.5 mM glucose, 2.5 mM CaCl_2_ and 1.3 mM MgSO_4_) and allowed to equilibrate for at least 1 h before recording. For recording, slices were placed in a recording chamber mounted on an Olympus BX51WI microscope equipped with infrared differential interference contrast optics (900 nm) and perfused with warmed (30–33 °C) oxygenated recording ACSF at a flow rate of 2 ml min^−1^.

*Field potential measurements*. Slices were perfused with modified recording ACSF (125 mM NaCl, 2.5 mM KCl, 0.8 mM NaH_2_PO_4_, 26 mM NaHCO_3_, 12.5 mM glucose, 4 mM MgCl_2_ and 4 mM CaCl_2_). A bipolar concentric electrode was placed in the DG granule cell layer to evoke fEPSPs. fEPSPs were recorded in the stratum lucidum of the CA3 region with glass micropipettes (3–4 MΩ) filled with recording ACSF. Data were acquired with a MultiClamp 700B amplifier (Molecular Devices) and WinLTP software (University of Bristol). The stimulation pulse width was 0.2 ms and the stimulation rate was 0.05 Hz throughout the experiment unless otherwise noted. Three responses were averaged for each data point. Mossy fibre fEPSPs were identified with the following criteria^65^: (1) negative waveform restricted to the stratum lucidum; (2) short onset latency (<5 ms); (3) fast time course (<10 ms); and (4) selective reduction by the group II metabotropic glutamate receptor agonist (2 S,2′R,3′R)-2-(2,3-dicarboxycyclopropyl)glycine (DCG-IV, 2 μM; Tocris Bioscience). Stimulation strength that elicited 30% of the maximum response was used to assess paired-pulse facilitation and LTP. Paired-pulse facilitation was induced with two stimulation pulses 50 ms apart, and the paired-pulse ratio was calculated by dividing the response elicited with the second pulse by the response elicited with the first pulse. LTP was induced with 125 stimulation pulses at 25 Hz. Data were analysed offline with Clampfit software (Molecular Devices). Slices in which baseline fEPSP responses fluctuated >20% were excluded from the analysis.

*Intrinsic excitability measurements*. Intrinsic excitability of DG granule cells was assessed by recording the frequency of action potentials in response to current stimulation (FI curve) during synaptic receptor blockade. To block AMPA, NMDA and GABA_A_ receptors, 20 μM 6,7-dinitroquinoxaline-2,3-dione, 100 μM DL-2-amino-5-phophonopentanoic acid (DL-APV) and 50 μM picrotoxin were added to recording ACSF (Tocris Bioscience). Patch pipettes with a tip resistance of 4–6 MΩ were filled with a solution consisting 20 mM KCl, 100 mM K-gluconate, 10 mM HEPES, 4 mM Mg-ATP, 0.3 mM Na-GTP, 10 mM Na-phosphocreatine, 0.2 mM EGTA and 0.2% biocytin (295 mOsm, pH 7.35). While the membrane potential of neurons was held at –75 mV, stimulating currents of different amplitudes were applied in a random sequence to generate an FI curve. To estimate the slopes of individual FI curves we fit a straight line between the current threshold and 100 (average frequency) or 300 pA (instantaneous frequency) above the current threshold. Data were analysed offline with a custom analysis programme in IGOR Pro software (WaveMetrics). Recordings from neurons that had a resting membrane potential >−55 mV or an access resistance >30 MΩ were excluded from analysis.

### Neuronal reconstruction and morphometry analysis

Before immunostaining, acute slices containing biocytin-filled dentate granule cells were fixed with 4% paraformaldehyde for 2 h at room temperature. Sections were maintained in solutions containing 0.25% Triton X-100 for the entire immunostaining protocol. Sections were blocked for 1 h at room temperature with 10% goat serum and incubated overnight at 4 °C in anti-GFP (1:500, MAB3580, Millipore) diluted in 3% goat serum. After PBS washing steps, biocytin-filled neurons were revealed, and the GFP signal was amplified by co-application of streptavidin-conjugated Alexa-594 (1:500, Invitrogen) and mouse Alexa 488 (1:500, Invitrogen) for 2 h at room temperature. After final washing steps, slices were mounted in Vectashield mounting medium with DAPI (Vector Laboratories).

Streptavidin-labelled neurons were visualized using epifluorescence and were imaged with a spectral confocal microscope (Nikon C1si) and EZ-C1 software. Whole dendritic trees were imaged with a Plan Fluor × 40 objective, 512 × 512 pixels, and in z-stacks of 0.5-μm steps. Whole cell bodies were imaged with a Plan × 100 objective and appropriate zoom, 512 × 512 pixels, and in z-stacks of 0.25-μm steps. Neuronal morphology was analysed with Fiji software. To estimate the size of cell bodies, z-stacks were flattened into maximum intensity projections, cell body outlines were traced and the total area of the tracings was quantified. To estimate the size of dendritic arbours, all dendrites of a given neuron were traced in three-dimensional with the Simple Neurite Tracer plugin, and their lengths were summed.

### Blind coding and statistical analysis

Investigators who obtained data were blinded to the disease state of human samples and to the genotype and treatment of mice and cell cultures. Sample sizes were chosen on the basis of pilot experiments and our experience with similar experiments. Statistical analyses were performed with Prism 6 (GraphPad) or R (R Development Core Team). Normal distribution of the data was verified with D'Agostino and Pearson's omnibus normality test. Differences between two means were assessed by unpaired or paired Student's *t*-test or, if the data were not normally distributed, with the Mann–Whitney *U*-test. Differences among multiple means were assessed by one-way or two-way analysis of variance or repeated-measures one-way analysis of variance, followed by Bonferroni's *post hoc* tests. Null hypotheses were rejected at the 0.05 level.

To analyse the data obtained in the acquisition phase of the MWM in greater detail, we estimated learning curves for each genotype-by-treatment pair using a Bayesian hierarchical model fit by Markov chain Monte Carlo implemented in the statistical programming language R^66^. For each pair, the likelihood included a 3-degrees-of-freedom natural cubic spline. For any given trial on a given day, we also included linearly time-varying effects of three dummy variables, indicating whether each observation was from the second, third or fourth trial of the day. These fixed effects were all given flat priors.

To account for the correlation among repeated observations, one random intercept and three time-spline terms were included for each mouse. For each of the model's four variance components (one for the random intercepts and one for each of the three random spline terms), we used a flat prior on the s.d. scale^67^. To account for censoring at 60 s, we used a latent variable approach^68^ that enabled Gibbs sampling of all parameters. To determine differences in learning strategies between the genotypes for a given treatment, we approximated *P* values by inverting 95% credible intervals, which represent the 2.5–97.5 percentiles of the posterior distribution.

To ensure the validity of our model-fitting algorithm, we started three Markov chain Monte Carlo chains from overdispersed initial values. After 2,500 iterations of burn-in, we obtained 2,500 additional samples from each chain. Convergence was achieved, with all chains having Gelman Rubin statistics^69^ of 1. We combined the three chains and thinned to obtain 5,000 iterations with which to do inference. Mixing yielded an average of 3,500 effectively independent samples^70^ from each chain.

## Additional information

**How to cite this article:** Suberbielle, E. *et al*. DNA repair factor BRCA1 depletion occurs in Alzheimer brains and impairs cognitive function in mice. *Nat. Commun*. 6:8897 doi: 10.1038/ncomms9897 (2015).

## Supplementary Material

Supplementary Information Supplementary Figures 1-14

## References

[b1] Jeppesen D. K., Bohr V. A. & Stevnsner T. DNA repair deficiency in neurodegeneration. Prog. Neurobiol. 94, 166–200 (2011). 21550379 10.1016/j.pneurobio.2011.04.013 PMC3123739

[b2] Madabhushi R., Pan L. & Tsai L. H. DNA damage and its links to neurodegeneration. Neuron 83, 266–282 (2014). 25033177 10.1016/j.neuron.2014.06.034 PMC5564444

[b3] Brasnjevic I., Hof P. R., Steinbusch H. W. & Schmitz C. Accumulation of nuclear DNA damage or neuron loss: molecular basis for a new approach to understanding selective neuronal vulnerability in neurodegenerative diseases. DNA Repair (Amst.) 7, 1087–1097 (2008). 18458001 10.1016/j.dnarep.2008.03.010 PMC2919205

[b4] Suberbielle E. . Physiologic brain activity causes DNA double-strand breaks in neurons, with exacerbation by amyloid-β. Nat. Neurosci. 16, 613–621 (2013). 23525040 10.1038/nn.3356 PMC3637871

[b5] Huang Y. & Mucke L. Alzheimer mechanisms and therapeutic strategies. Cell 148, 1204–1222 (2012). 22424230 10.1016/j.cell.2012.02.040 PMC3319071

[b6] Cissé M. . Reversing EphB2 depletion rescues cognitive functions in Alzheimer model. Nature 469, 47–52 (2011). 21113149 10.1038/nature09635 PMC3030448

[b7] Palop J. J. . Neuronal depletion of calcium-dependent proteins in the dentate gyrus is tightly linked to Alzheimer's disease-related cognitive deficits. Proc. Natl Acad. Sci. USA 100, 9572–9577 (2003). 12881482 10.1073/pnas.1133381100 PMC170959

[b8] Lesne S. E. . Brain amyloid-β oligomers in ageing and Alzheimer's disease. Brain 136, 1383–1398 (2013). 23576130 10.1093/brain/awt062 PMC3634198

[b9] Huen M. S., Sy S. M. & Chen J. BRCA1 and its toolbox for the maintenance of genome integrity. Nat. Rev. Mol. Cell Biol. 11, 138–148 (2010). 20029420 10.1038/nrm2831 PMC3899800

[b10] Jeon G. S. . Deregulation of BRCA1 leads to impaired spatiotemporal dynamics of gamma-H2AX and DNA damage responses in Huntington's disease. Mol. Neurobiol. 45, 550–563 (2012). 22580959 10.1007/s12035-012-8274-9 PMC4642996

[b11] Thompson L. H. Recognition, signaling, and repair of DNA double-strand breaks produced by ionizing radiation in mammalian cells: the molecular choreography. Mutat. Res. 751, 158–246 (2012). 22743550 10.1016/j.mrrev.2012.06.002

[b12] Pulvers J. N. & Huttner W. B. Brca1 is required for embryonic development of the mouse cerebral cortex to normal size by preventing apoptosis of early neural progenitors. Development 136, 1859–1868 (2009). 19403657 10.1242/dev.033498

[b13] Evans T. A. . BRCA1 may modulate neuronal cell cycle re-entry in Alzheimer disease. Int. J. Med. Sci. 4, 140–145 (2007). 17505559 10.7150/ijms.4.140 PMC1868658

[b14] Silva A. R. . Repair of oxidative DNA damage, cell-cycle regulation and neuronal death may influence the clinical manifestation of Alzheimer's disease. PLoS ONE 9, e99897 (2014). 24936870 10.1371/journal.pone.0099897 PMC4061071

[b15] Ibrahim N. . BRCA1-associated epigenetic regulation of p73 mediates an effector pathway for chemosensitivity in ovarian carcinoma. Cancer Res. 70, 7155–7165 (2010). 20807817 10.1158/0008-5472.CAN-10-0668 PMC2940979

[b16] Chin J. . Fyn kinase induces synaptic and cognitive impairments in a transgenic mouse model of Alzheimer's disease. J. Neurosci. 25, 9694–9703 (2005). 16237174 10.1523/JNEUROSCI.2980-05.2005 PMC6725734

[b17] Collins A. R. The comet assay for DNA damage and repair: principles, applications, and limitations. Mol. Biotechnol. 26, 249–261 (2004). 15004294 10.1385/MB:26:3:249

[b18] Kempermann G., Gast D., Kronenberg G., Yamaguchi M. & Gage F. H. Early determination and long-term persistence of adult-generated new neurons in the hippocampus of mice. Development 130, 391–399 (2003). 12466205 10.1242/dev.00203

[b19] Mucke L. . High-level neuronal expression of Aβ_1-42_ in wild-type human amyloid protein precursor transgenic mice: synaptotoxicity without plaque formation. J. Neurosci. 20, 4050–4058 (2000). 10818140 10.1523/JNEUROSCI.20-11-04050.2000 PMC6772621

[b20] Kobayashi D. T. & Chen K. S. Behavioral phenotypes of amyloid-based genetically modified mouse models of Alzheimer's disease. Genes Brain Behav. 4, 173–196 (2005). 15810905 10.1111/j.1601-183X.2005.00124.x

[b21] Clark R. E. & Martin S. J. Interrogating rodents regarding their object and spatial memory. Curr. Opin. Neurobiol. 15, 593–598 (2005). 16150589 10.1016/j.conb.2005.08.014

[b22] Choudhury A. D., Xu H. & Baer R. Ubiquitination and proteasomal degradation of the BRCA1 tumor suppressor is regulated during cell cycle progression. J. Biol. Chem. 279, 33909–33918 (2004). 15166217 10.1074/jbc.M403646200

[b23] Hardingham G. E., Fukunaga Y. & Bading H. Extrasynaptic NMDARs oppose synaptic NMDARs by triggering CREB shut-off and cell death pathways. Nat. Neurosci. 5, 405–414 (2002). 11953750 10.1038/nn835

[b24] Madabhushi R. . Activity-induced DNA breaks govern the expression of neuronal early-response genes. Cell 161, 1592–1605 (2015). 26052046 10.1016/j.cell.2015.05.032 PMC4886855

[b25] Bucholtz N. & Demuth I. DNA-repair in mild cognitive impairment and Alzheimer's disease. DNA Repair (Amst.) 12, 811–816 (2013). 23919922 10.1016/j.dnarep.2013.07.005

[b26] Khurana S. . A macrohistone variant links dynamic chromatin compaction to BRCA1-dependent genome maintenance. Cell Rep. 8, 1049–1062 (2014). 25131201 10.1016/j.celrep.2014.07.024 PMC4154351

[b27] Ayrapetov M. K., Gursoy-Yuzugullu O., Xu C., Xu Y. & Price B. D. DNA double-strand breaks promote methylation of histone H3 on lysine 9 and transient formation of repressive chromatin. Proc. Natl Acad. Sci. USA 111, 9169–9174 (2014). 24927542 10.1073/pnas.1403565111 PMC4078803

[b28] Small S. A. Isolating pathogenic mechanisms embedded within the hippocampal circuit through regional vulnerability. Neuron 84, 32–39 (2014). 25277453 10.1016/j.neuron.2014.08.030 PMC4185396

[b29] Gallagher M. & Koh M. T. Episodic memory on the path to Alzheimer's disease. Curr. Opin. Neurobiol. 21, 929–934 (2011). 22079495 10.1016/j.conb.2011.10.021 PMC3254732

[b30] Bakker A. . Reduction of hippocampal hyperactivity improves cognition in amnestic mild cognitive impairment. Neuron 74, 467–474 (2012). 22578498 10.1016/j.neuron.2012.03.023 PMC3351697

[b31] Robinson J. L. . Perforant path synaptic loss correlates with cognitive impairment and Alzheimer's disease in the oldest-old. Brain 137, 2578–2587 (2014). 25012223 10.1093/brain/awu190 PMC4132652

[b32] Wakabayashi K., Honer W. G. & Masliah E. Synapse alterations in the hippocampal-entorhinal formation in Alzheimer's disease with and without Lewy body disease. Brain Res. 667, 24–32 (1994). 7895080 10.1016/0006-8993(94)91709-4

[b33] Kuroda H., Kutner R. H., Bazan N. G. & Reiser J. A comparative analysis of constitutive and cell-specific promoters in the adult mouse hippocampus using lentivirus vector-mediated gene transfer. J. Gene Med. 10, 1163–1175 (2008). 18773500 10.1002/jgm.1249

[b34] Kheirbek M. A. . Differential control of learning and anxiety along the dorsoventral axis of the dentate gyrus. Neuron 77, 955–968 (2013). 23473324 10.1016/j.neuron.2012.12.038 PMC3595120

[b35] Arendt T., Bruckner M. K., Bigl V. & Marcova L. Dendritic reorganisation in the basal forebrain under degenerative conditions and its defects in Alzheimer's disease. III. The basal forebrain compared with other subcortical areas. J. Comp. Neurol. 351, 223–246 (1995). 7699112 10.1002/cne.903510204

[b36] Moolman D. L., Vitolo O. V., Vonsattel J. P. & Shelanski M. L. Dendrite and dendritic spine alterations in Alzheimer models. J. Neurocytol. 33, 377–387 (2004). 15475691 10.1023/B:NEUR.0000044197.83514.64

[b37] Ferrer I. Neurons and their dendrites in frontotemporal dementia. Dement. Geriatr. Cogn. Disord. 10, 55–60 (1999). 10436342 10.1159/000051214

[b38] Zaja-Milatovic S. . Dendritic degeneration in neostriatal medium spiny neurons in Parkinson disease. Neurology 64, 545–547 (2005). 15699393 10.1212/01.WNL.0000150591.33787.A4

[b39] Vetter P., Roth A. & Hausser M. Propagation of action potentials in dendrites depends on dendritic morphology. J. Neurophysiol. 85, 926–937 (2001). 11160523 10.1152/jn.2001.85.2.926

[b40] Siskova Z. . Dendritic structural degeneration is functionally linked to cellular hyperexcitability in a mouse model of Alzheimer's disease. Neuron 84, 1023–1033 (2014). 25456500 10.1016/j.neuron.2014.10.024

[b41] Otto C. . Impairment of mossy fiber long-term potentiation and associative learning in pituitary adenylate cyclase activating polypeptide type I receptor-deficient mice. J. Neurosci. 21, 5520–5527 (2001). 11466423 10.1523/JNEUROSCI.21-15-05520.2001 PMC6762677

[b42] Dumas T. C., Powers E. C., Tarapore P. E. & Sapolsky R. M. Overexpression of calbindin D_28k_ in dentate gyrus granule cells alters mossy fiber presynaptic function and impairs hippocampal-dependent memory. Hippocampus 14, 701–709 (2004). 15318329 10.1002/hipo.10210

[b43] Busche M. A. . Critical role of soluble amyloid-β for early hippocampal hyperactivity in a mouse model of Alzheimer's disease. Proc. Natl Acad. Sci. USA 109, 8740–8745 (2012). 22592800 10.1073/pnas.1206171109 PMC3365221

[b44] Vossel K. A. . Seizures and epileptiform activity in the early stages of Alzheimer disease. JAMA Neurol. 70, 1158–1166 (2013). 23835471 10.1001/jamaneurol.2013.136 PMC4013391

[b45] Bannerman D. M. . Hippocampal synaptic plasticity, spatial memory and anxiety. Nat. Rev. Neurosci. 15, 181–192 (2014). 24552786 10.1038/nrn3677

[b46] D'Hooge R. & De Deyn P. P. Applications of the Morris water maze in the study of learning and memory. Brain Res. Rev. 36, 60–90 (2001). 11516773 10.1016/s0165-0173(01)00067-4

[b47] Moser M. B., Moser E. I., Forrest E., Andersen P. & Morris R. G. Spatial learning with a minislab in the dorsal hippocampus. Proc. Natl Acad. Sci. USA 92, 9697–9701 (1995). 7568200 10.1073/pnas.92.21.9697 PMC40869

[b48] Gu Y. . Optical controlling reveals time-dependent roles for adult-born dentate granule cells. Nat. Neurosci. 15, 1700–1706 (2012). 23143513 10.1038/nn.3260 PMC3509272

[b49] McConnell M. J. . Mosaic copy number variation in human neurons. Science 342, 632–637 (2013). 24179226 10.1126/science.1243472 PMC3975283

[b50] Reilly M. T., Faulkner G. J., Dubnau J., Ponomarev I. & Gage F. H. The role of transposable elements in health and diseases of the central nervous system. J. Neurosci. 33, 17577–17586 (2013). 24198348 10.1523/JNEUROSCI.3369-13.2013 PMC3818539

[b51] Shi Y. & Whetstine J. R. Dynamic regulation of histone lysine methylation by demethylases. Mol. Cell 25, 1–14 (2007). 17218267 10.1016/j.molcel.2006.12.010

[b52] Day J. J. & Sweatt J. D. Epigenetic mechanisms in cognition. Neuron 70, 813–829 (2011). 21658577 10.1016/j.neuron.2011.05.019 PMC3118503

[b53] Jiang G. . BRCA1-Ku80 protein interaction enhances end-joining fidelity of chromosomal double-strand breaks in the G1 phase of the cell cycle. J. Biol. Chem. 288, 8966–8976 (2013). 23344954 10.1074/jbc.M112.412650 PMC3610969

[b54] Dohrn L., Salles D., Siehler S. Y., Kaufmann J. & Wiesmuller L. BRCA1-mediated repression of mutagenic end-joining of DNA double-strand breaks requires complex formation with BACH1. Biochem. J. 441, 919–926 (2012). 22032289 10.1042/BJ20110314

[b55] Wang H. C., Chou W. C., Shieh S. Y. & Shen C. Y. Ataxia telangiectasia mutated and checkpoint kinase 2 regulate BRCA1 to promote the fidelity of DNA end-joining. Cancer Res. 66, 1391–1400 (2006). 16452194 10.1158/0008-5472.CAN-05-3270

[b56] Xiong X. . 53BP1 promotes microhomology-mediated end-joining in G1-phase cells. Nucleic Acids Res. 43, 1659–1670 (2015). 25586219 10.1093/nar/gku1406 PMC4330367

[b57] Badie S. . BRCA1 and CtIP promote alternative non-homologous end-joining at uncapped telomeres. EMBO J. 34, 410–424 (2015). 25582120 10.15252/embj.201488947 PMC4339125

[b58] Bothmer A. . 53BP1 regulates DNA resection and the choice between classical and alternative end joining during class switch recombination. J. Exp. Med. 207, 855–865 (2010). 20368578 10.1084/jem.20100244 PMC2856023

[b59] Albert M. S. . The diagnosis of mild cognitive impairment due to Alzheimer's disease: recommendations from the National Institute on Aging-Alzheimer's Association workgroups on diagnostic guidelines for Alzheimer's disease. Alzheimers Dement. 7, 270–279 (2011). 21514249 10.1016/j.jalz.2011.03.008 PMC3312027

[b60] McKhann G. M. . The diagnosis of dementia due to Alzheimer's disease: recommendations from the National Institute on Aging-Alzheimer's Association workgroups on diagnostic guidelines for Alzheimer's disease. Alzheimers Dement. 7, 263–269 (2011). 21514250 10.1016/j.jalz.2011.03.005 PMC3312024

[b61] Hyman B. T. . National Institute on Aging-Alzheimer's Association guidelines for the neuropathologic assessment of Alzheimer's disease. Alzheimers Dement. 8, 1–13 (2012). 22265587 10.1016/j.jalz.2011.10.007 PMC3266529

[b62] Harris J. A. . Transsynaptic progression of amyloid-β-induced neuronal dysfunction within the entorhinal-hippocampal network. Neuron 68, 428–441 (2010). 21040845 10.1016/j.neuron.2010.10.020 PMC3050043

[b63] Thakur S. . Localization of BRCA1 and a splice variant identifies the nuclear localization signal. Mol. Cell Biol. 17, 444–452 (1997). 8972225 10.1128/mcb.17.1.444 PMC231769

[b64] Ting J. T., Daigle T. L., Chen Q. & Feng G. Acute brain slice methods for adult and aging animals: application of targeted patch clamp analysis and optogenetics. Methods Mol. Biol. 1183, 221–242 (2014). 25023312 10.1007/978-1-4939-1096-0_14 PMC4219416

[b65] Calixto E., Thiels E., Klann E. & Barrionuevo G. Early maintenance of hippocampal mossy fiber--long-term potentiation depends on protein and RNA synthesis and presynaptic granule cell integrity. J. Neurosci. 23, 4842–4849 (2003). 12832506 10.1523/JNEUROSCI.23-12-04842.2003 PMC6741163

[b66] R Development Core Team. R: A language and environment for statistical computing R Foundation for Statistical Computing (2013).

[b67] Gelman A. Prior distributions for variance parameters in hierarchical models. Bayesian Anal. 1, 515–534 (2006).

[b68] Gelman A. & Hill J. in Data Analysis Using Regression and Multilevel/Hierarchical Models 406–408Cambridge University Press (2007).

[b69] Gelman A. & Rubin D. B. Inference from iterative simulation using multiple sequences. Stat. Sci. 7, 457–472 (1992).

[b70] Plummer M., Best N., Cowles K. & Vines K. CODA: Convergence Diagnosis and Output Analysis for MCMC. R News 6, 7–11 (2006).

