# Supplementary material for: DNA repair factor BRCA1 depletion occurs in Alzheimer brains and impairs cognitive function in mice
Source: Nat Commun. 2015 Nov 30;6:8897. doi: 10.1038/ncomms9897 (PMC4674776; doi:10.1038/ncomms9897)
Supplement: Supplementary Information — Supplementary Figures 1-14 [file ncomms9897-s1.pdf]

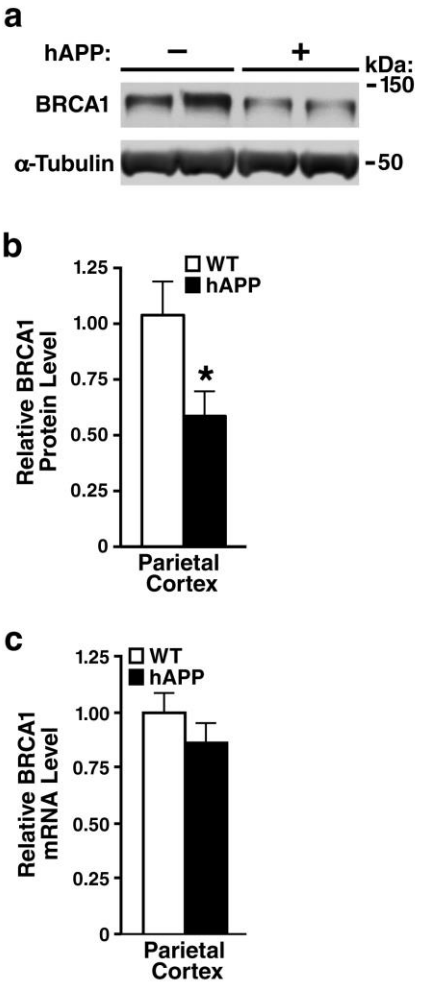

Supplementary Figure 1 | hAPP-J20 mice have reduced BRCA1 protein levels but normal BRCA1 mRNA levels in parietal cortex. (a,b) BRCA1 protein levels in the parietal cortex of WT) and hAPP-J20 mice were determined by western blot analysis. **(a)** Representative western blot. **(b)** Quantitation of western blot signals. The average BRCA1 to α-tubulin ratio in WT mice was arbitrarily defined as 1.0. *n* = 15–20 mice per genotype. Age, 4–8 months. Each lane contained a sample from a differnt mouse. **(c)** Levels of BRCA1 mRNA were assessed by RT-qPCR. The average BRCA1 to GAPDH mRNA ratio in WT mice was arbitrarily defined as 1.0. *n* = 11–13 mice per genotype. Age, 4–6 months. $^*P < 0.05$ vs. WT by *t*-test (with Welch correction). Values in bar graphs are means ± s.e.m.

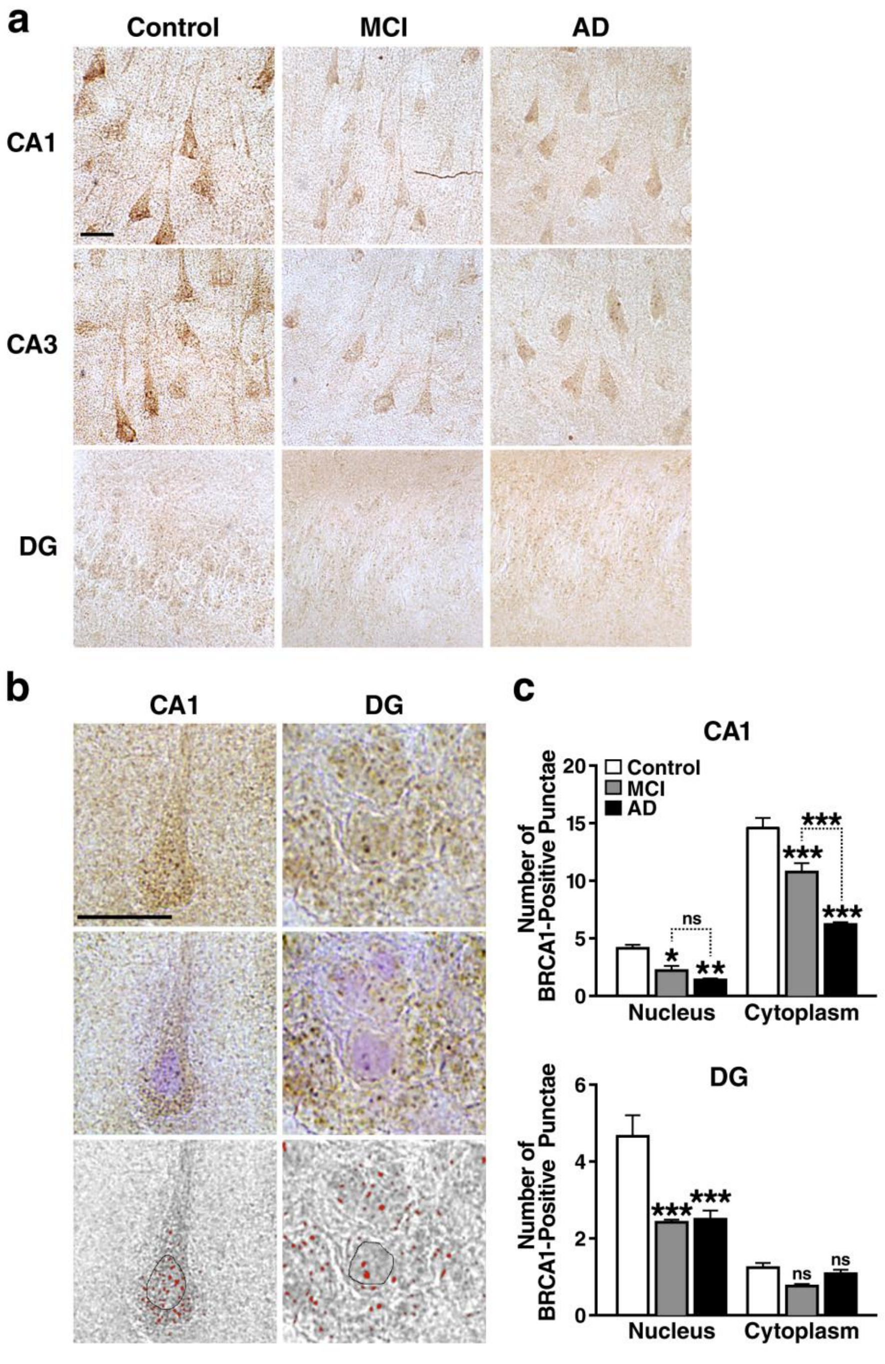

Supplementary Figure 2 | **Neuronal BRCA1 reduction in the hippocampus of MCI and AD patients. (a)** Lower magnification images of neuronal BRCA1 immunoreactivities corresponding to Figure 2a. Scale bar, 20 μm. **(b,c)** Quantitation of BRCA1 immunoreactivity in CA1 and DG

neurons. **(b)** Images showing neurons immunoperoxidase stained for BRCA1 (top), the same neurons counterstained with haematoxylin (middle), and corresponding processed gray-scale renditions (bottom). Immunoreactive punctae (red dots) were counted semiautomatically after the image threshold was adjusted with the quantitation software and the nuclei (black circles) were manually delineated. Scale bar, 20 μm. **(c)** Average number of BRCA1-immunoreactive punctae detected in the nucleus or cytoplasm per cell, as determined by analysis of 10 cells per case. n = 5 cases/group. *P < 0.05, **P < 0.01, ***P < 0.001 vs. leftmost bar or as indicated by brackets (Bonferroni test). Bars represent means ± SEM.

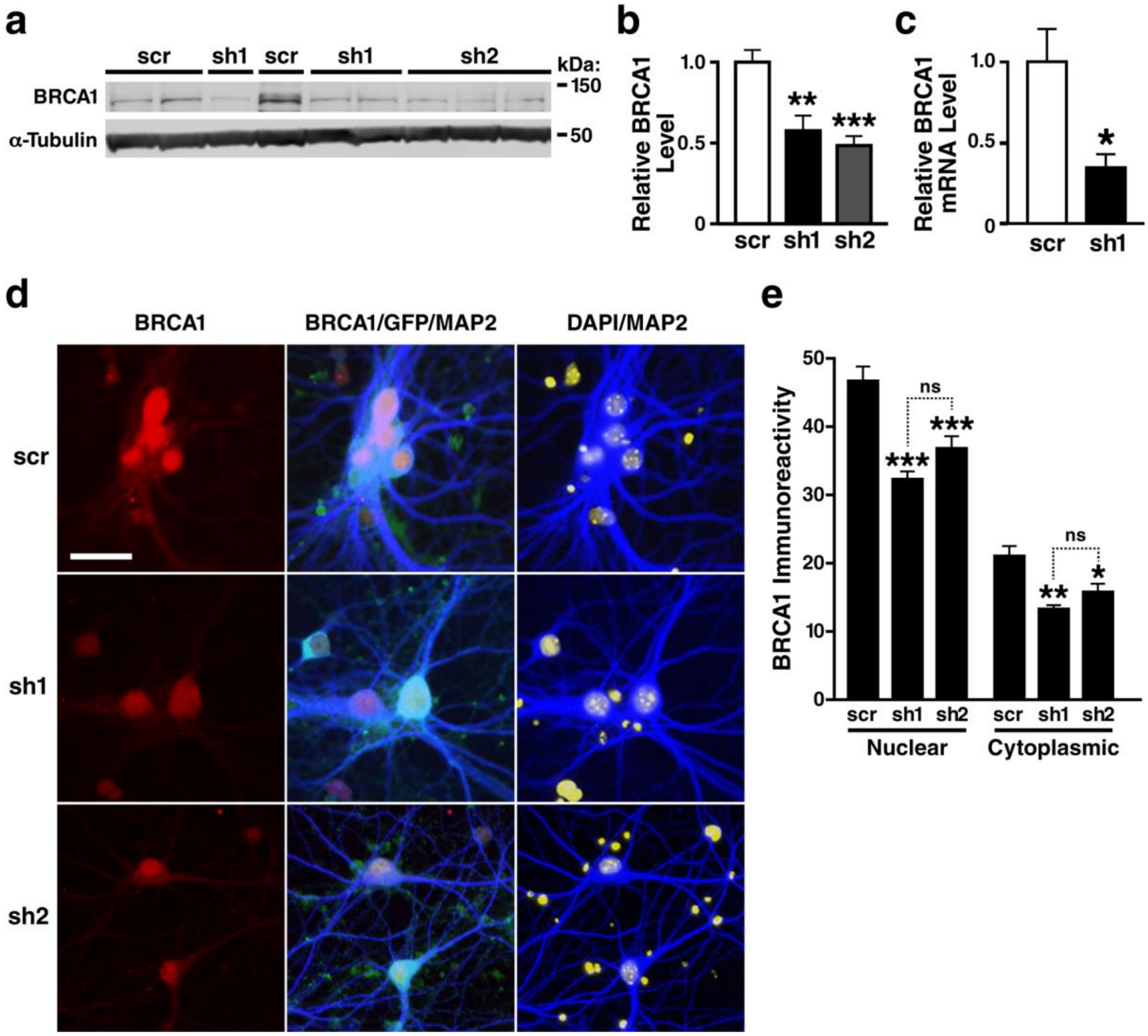

Supplementary Figure 3 | shRNA-mediated knockdown of BRCA1 in primary neuronal cultures and in the DG of mice. Two different shRNAs (sh) targeting BRCA1 mRNA (#1 and #2) or a scrambled (scr) shRNA were incorporated separately into a lentiviral vector encoding EGFP. **(a–e)** Cultures of primary hippocampal neurons from WT mice were infected with LV-sh1-GFP (sh1), LV-sh2-GFP (sh2) or LV-scr-GFP (scr) on DIV 7 and analyzed for BRCA1 expression by western blotting (**a,b**), RT-qPCR (**c**), or immunostaining and fluorescence microscopy (**d**) 7 days later. **(a)** Representative western blot. **(b)** Quantitation of western blot signals. *n* = 4–7 wells/condition from 3 independent experiments. **(c)** BRCA1 mRNA levels. *n* = 10–12 wells/condition from 3 independent experiments. **(d)** Photomicrographs of neuronal

cultures transduced with the indicated viral vectors, fixed, immunostained for BRCA1 (red), GFP (green), and the neuronal marker MAP2 (blue), and labeled with DAPI (yellow). Viral transduction of neurons is reflected by colocalization of GFP and MAP2 (colocalization is indicated by turquoise color) and indicated with white arrowheads (middle panels). Note the lower intensity of BRCA1 signals in the cultures transduced with sh1 or sh2. Multiplicity of infection: 0.75 TU per cell. Scale bar, 20 μm. (**e**) Quantitation of nuclear and cytoplasmic BRCA1 levels in individual neurons. For each shRNA, 44–47 GFP/MAP2-positive cells were selected randomly from three replicate cultures, and BRCA1 immunoreactivity in the nucleus versus cytoplasm was quantitated by densitometry using the 8-bit gray-scale format of the red channel. Each value was normalized to the background of cultures not labeled with primary antibody. The average ratio of BRCA1 to GAPDH (**c**) or α-tubulin (**b**) in scr-infected cultures was defined as 1.0. $*P < 0.05$, $**P < 0.01$, $***P < 0.001$ vs. WT/scr (**b**) or scr for the same compartment (**e**) by Bonferroni test or by unpaired *t*-test (**c**). Bars represent means ± s.e.m.

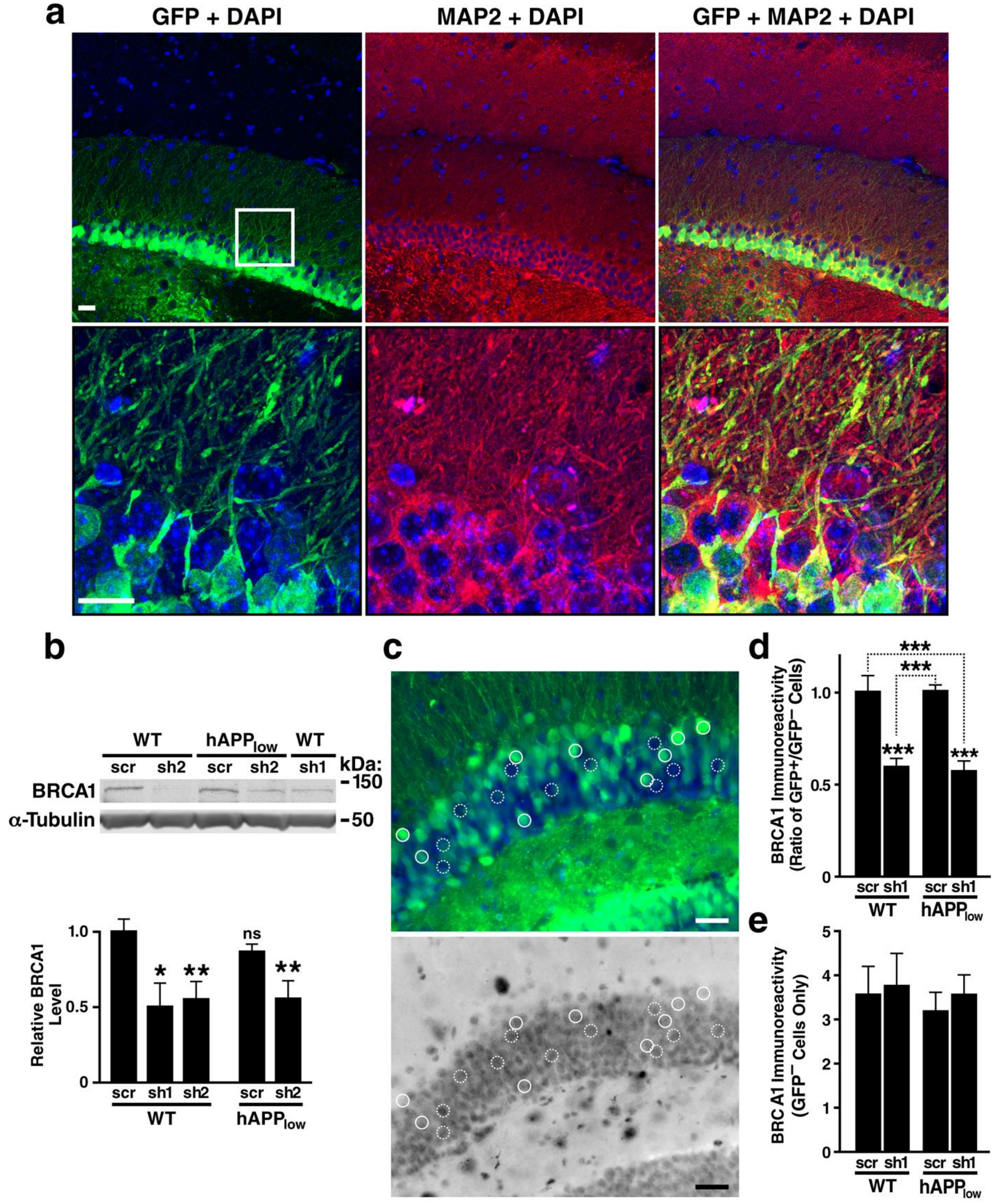

Supplementary Figure 4 | **Knockdown of BRCA1 in dentate granule cells of mice.** WT and $hAPP_{low}$ mice received bilateral stereotaxic injections of scr or sh1 or sh2 into the DG at 2.7–3.9 months (**a,b**) or 1–2 months (**c–e**) of age and were analyzed 2.5 to 3 months later. **(a)**

Transduction of dentate granule cells by lentiviral constructs expressing anti-BRCA1 shRNAs. DG sections from an sh2-injected $hAPP_{low}$ mouse were immunostained for GFP (green) and MAP2 (red) and co-labeled with DAPI (blue). Confocal fluorescence micrographs show colocalization of GFP (indicating shRNA-expressing neurons) and the neuron-specific marker MAP2. The images in the lower row show higher magnification views of the area marked with the white box in the image above. Scale bars: 20 µm. **(b)** The levels of BRCA1 in the DG were assessed by western blot analysis. Representative western blot and quantitation of western blot signals. n = 4–6 mice per condition. In western blots, each lane contained a sample from a different mouse. The average ratio of BRCA1 to α-tubulin in scr-infected WT mice was defined as 1.0. **(c)** Approach to quantitating BRCA1 levels in individual neurons *in vivo*. The top panel shows a fluorescence microscopic image of a DG section from an sh1-injected WT mouse immunostained for GFP (green) and BRCA1 (red, not shown) and co-labeled with DAPI (blue). To estimate BRCA1 levels in individual cells, 60 GFP-positive (solid white ovals) and 60 GFP-negative (stippled white ovals) granule cells in two triple-labeled sections per mouse were selected randomly on the basis of inspection of images showing only GFP and DAPI signals (top panel), followed by densitometric quantitation of BRCA1 immunoreactivity in each selected area on the corresponding 8-bit gray-scale format of the red channel (bottom panel). Scale bars, 20 μm. **(d)** Ratio of relative BRCA1 immunoreactivities in GFP-positive vs. GFP-negative neurons. The average ratio in scr-infected WT mice was defined as 1.0. n = 4–6 mice per genotype and treatment. **(e)** BRCA1 immunoreactivities in GFP-negative neurons. *$P < 0.05$, **$P < 0.01$, ***$P < 0.001$ vs. WT/scr (**b**) or vs. scr of the same genotype (**d**) or as indicated by brackets by Bonferroni test. Bars represent means ± s.e.m.

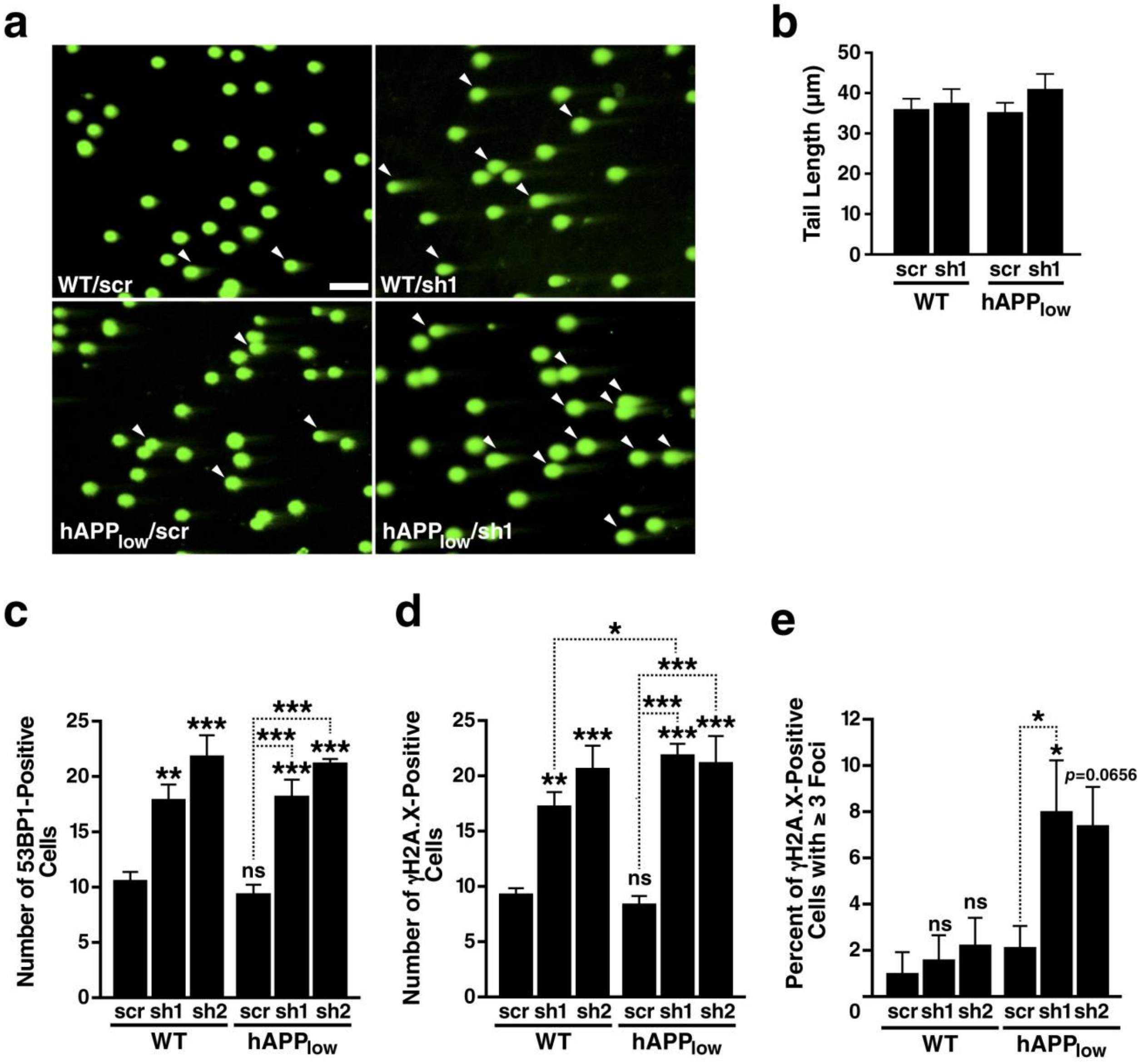

Supplementary Figure 5 | **Knockdown of BRCA1 increases DNA fragmentation in the DG of mice.** WT and $hAPP_{low}$ mice received bilateral stereotaxic injections of scr, sh1, or sh2 into the DG at 1–2 months (**a**,**b**), or 2.7–3.9 months (**c,e**) of age and were analyzed 3 months later. **(a,b)** Cell nuclei isolated from DG homogenates were assessed for DSB levels by comet assay carried out at neutral pH. **(a)** Representative images of cell nuclei from WT and $hAPP_{low}$ mice injected with scr or sh1. White arrowheads point to nuclei with comet tails, which reflect DNA fragmentation. Scale bar, 20 μm. **(b)** The severity of DNA fragmentation was assessed by

measuring the length of the comet tail for each nucleus that had a tail. For each mouse, 450–600 nuclei in 2–3 fields were inspected and scored (*n* = 3–5 mice per genotype and treatment). **(c–e)** Comparisons of DSB levels induced by sh1- versus sh2-mediated BRCA1 reduction. Brain sections were immunostained for 53BP1 (**c**) or γH2A.X (**d**), and dentate granule cells with 53BP1- or γH2A.X-positive foci were counted in two sections per mouse. (**c**) Number of granule cells per section with 53BP1-positive foci. *n* = 4–15 mice per genotype and treatment. (**d**) Number of granule cells per section with γH2A.X-positive foci. *n* = 4–6 mice per genotype and treatment. (**e**) Percentage of focus-bearing cells with ≥3 γH2A.X foci. *n* = 4–6 mice per genotype and treatment. $^{*}P < 0.05$, $^{**}P < 0.01$, $^{***}P < 0.001$ vs. WT/scr or as indicated by brackets (Bonferroni test). ns, not significant. Bars represent means ± s.e.m.

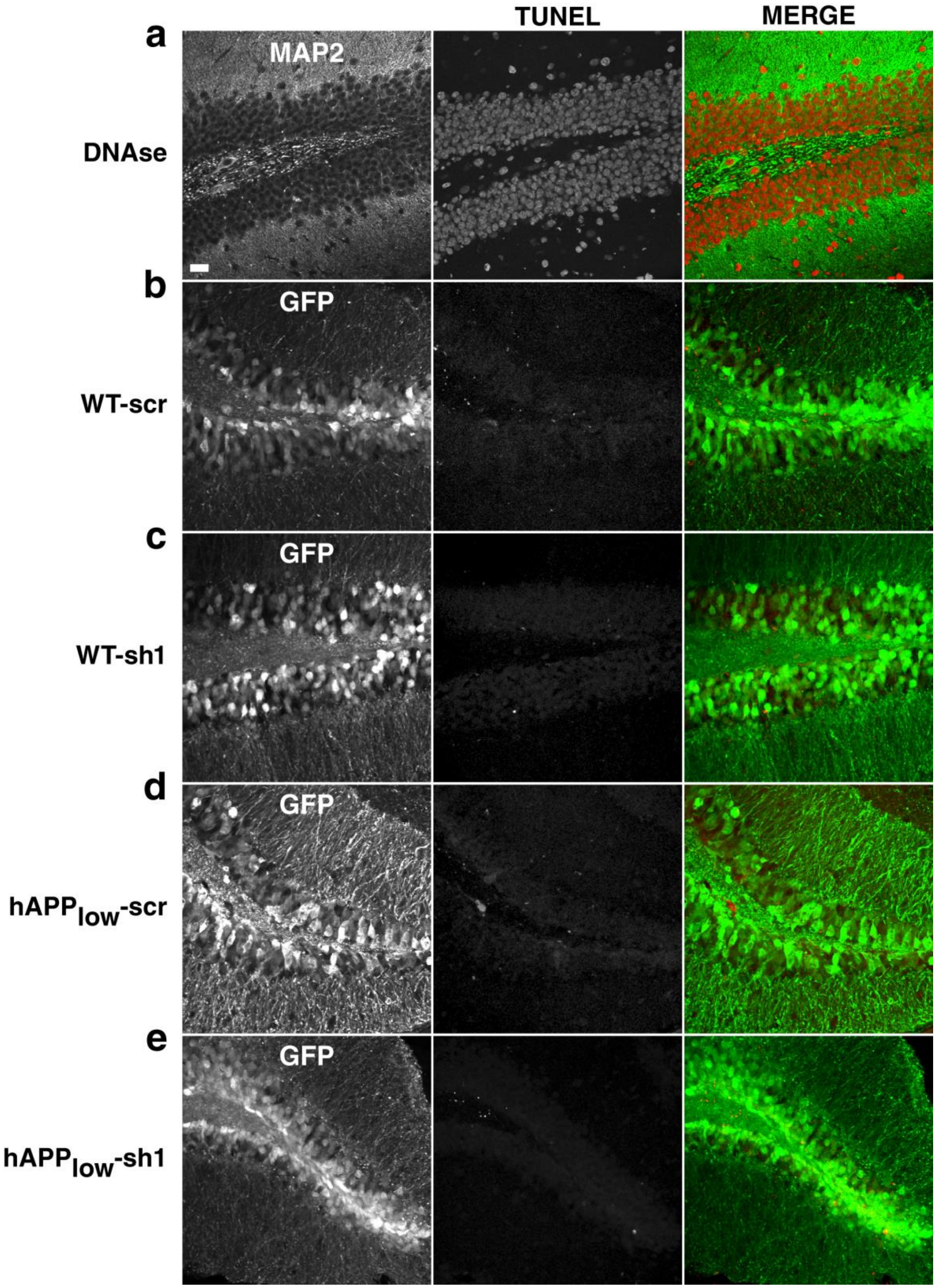

Supplementary Figure 6 | Knockdown of BRCA1 does not cause neuronal apoptosis in the DG of mice. WT and hAPP$_{low}$ mice received bilateral stereotaxic injections of LV-Scr-GFP

(scr) or LV-shBRCA1-GFP (sh1) into the DG at 1–2 months of age and were analyzed 3 months later. **(a–e)** After TUNEL staining (red or as indicated), coronal brain sections were immunostained for MAP2 (**a**) or GFP (**b–e**) (green or as indicated) and imaged by confocal microscopy. Photomicrographs show portions of the DG. Sections pretreated with DNAse (**a**) served as a positive control and had many TUNEL-positive neuronal nuclei. Note the absence or paucity of TUNEL/GFP double-labeled neurons in sh1-injected mice (**c–e**). Similar results were obtained in an additional 3 mice per genotype and treatment (data not shown). Scale bar, 20 μm. Bars represent means ± s.e.m.

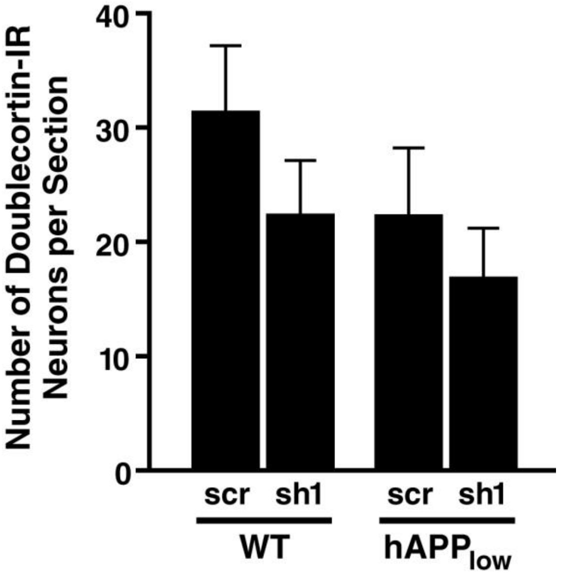

Supplementary Figure 7 | **Knockdown of BRCA1 does not significantly lower the number of newly born granule cells in the DG of mice.** WT and $hAPP_{low}$ mice received bilateral stereotaxic injections of LV-Scr-GFP (scr) or LV-shBRCA1-GFP (sh1) into the DG at 1–2 months of age and were analyzed 3 months later. The number of newly born granule cells in the DG was determined by immunostaining of coronal brain sections for doublecortin and counting immunoreactive (IR) neurons in 7 sections per mouse. The average number of IR neurons per section is shown (n = 10–13 mice per genotype and treatment). Two-way ANOVA revealed no significant differences among the groups. Bars represent means ± s.e.m.

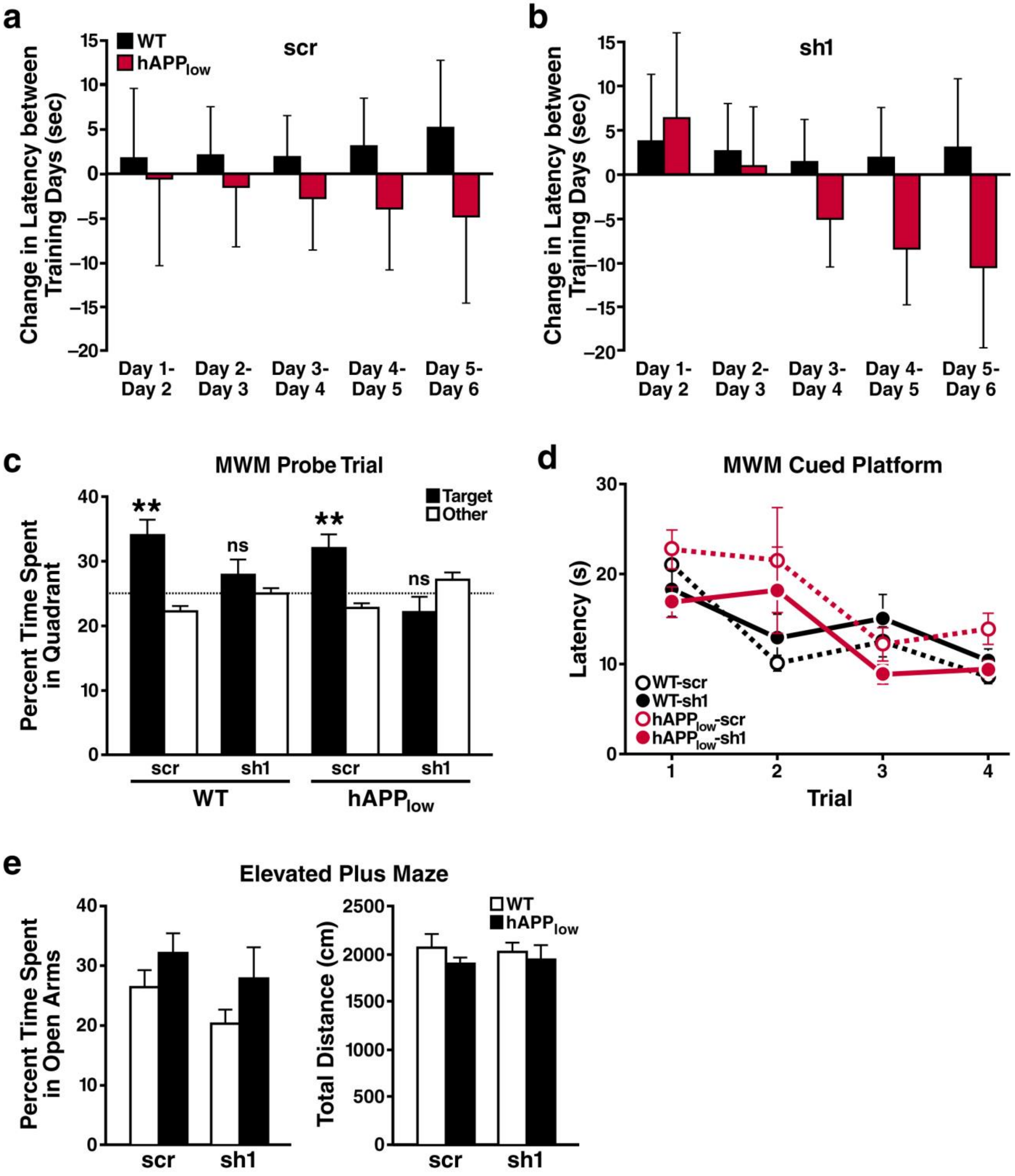

Supplementary Figure 8 | Knockdown of BRCA1 in the DG Impairs spatial, but not cued, learning/memory in the MWM and does not alter performance in the elevated plus maze. WT and $hAPP_{low}$ mice received bilateral stereotaxic injections of scr or sh1 into the DG at 1–2

months of age. They were tested in the Morris water maze (MWM) (**a–d**) and the elevated plus maze (**e**) 1 month later. **(a,b)** Change in escape latency between training days (latency of the first trial of each day minus mean latency of the last two trials on the preceding day) in mice injected with scr (**a**) or sh1 (**b**). *n* = 18–28 per genotype and treatment. **(a)** Scr-injected WT and $hAPP_{low}$ mice showed no significant differences in day-to-day changes in escape latency (P = 0.98, by Bayesian modeling), although $hAPP_{low}$ mice showed trends toward less improvement (no positive values) and more setbacks (all negative values). **(b)** Sh1-injected $hAPP_{low}$ mice showed greater day-to-day setbacks than sh1-injected WT mice during the last three intervals analyzed (P = 0.02, by Bayesian model). **(c)** Percent of time mice spent in the target quadrant vs. the three other quadrants during a probe trial 24 h after the last training trial in the hidden-platform component of the MWM. *n* = 18–28 per genotype and treatment. **(d)** Learning curves of mice tested in the cued platform component of the MWM (two trials per day on two consecutive days). *n* = 6–12 mice per genotype and treatment. Bayesian modeling revealed no significant differences among groups. Mice from two of the three cohorts tested in the hidden platform component of the MWM (Fig. 3) subsequently underwent cued platform testing. **(e)** Proportion of time mice spent in the open vs. closed arms of an elevated plus maze during the first 5 min of testing (left) and the total distance the mice traveled in the maze during the first 10 min of testing (right). Two-way ANOVA revealed no significant effect of genotype or treatment. *n* = 10–13 mice per genotype and treatment. **P < 0.01 vs. chance (dotted line) by one-sample *t*-test. ns, not significant. Values are means ± 95% CI (**a,b**) or means ± s.e.m. (**c–e**).

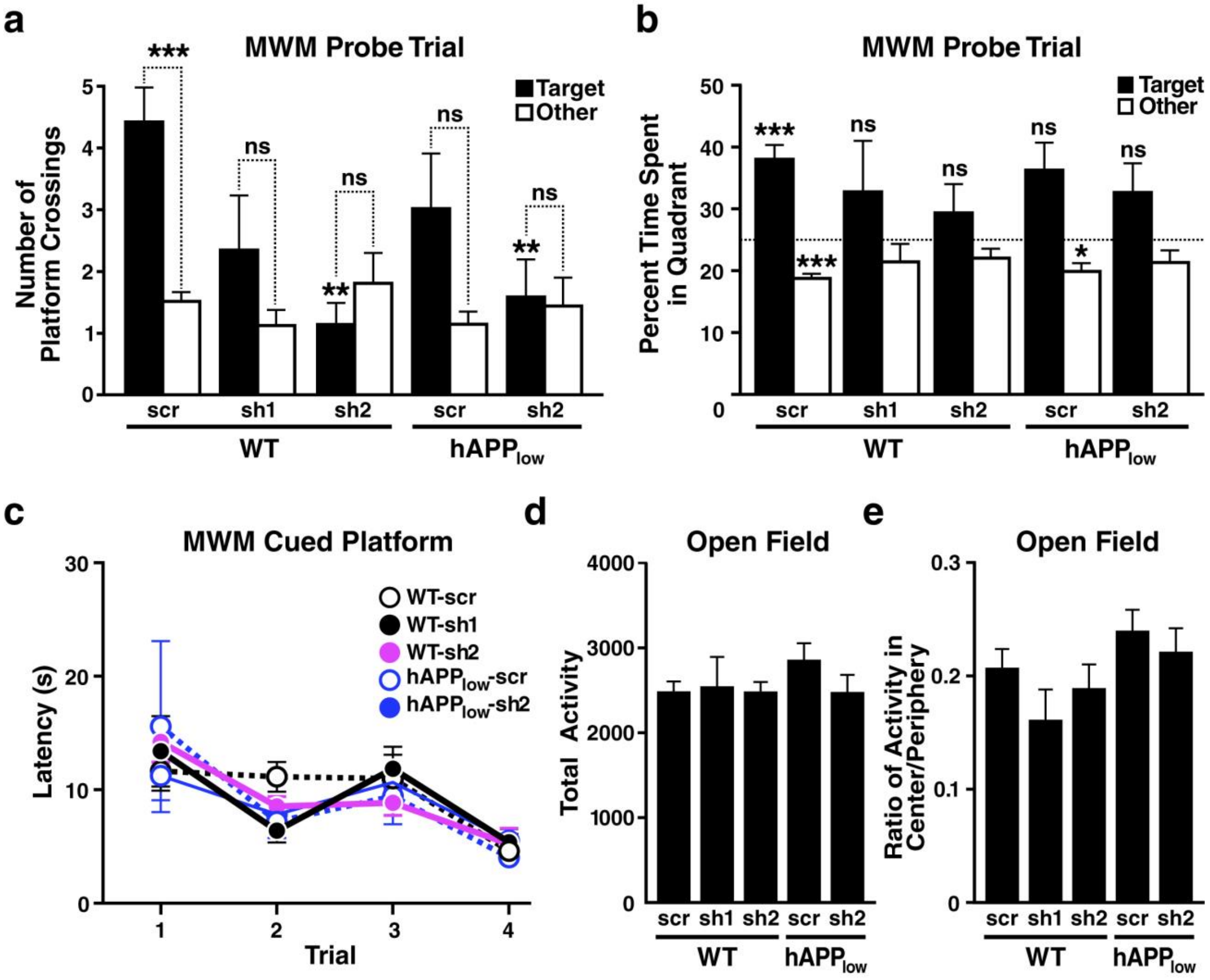

Supplementary Figure 9 | **Knockdown of BRCA1 with 2 shRNA in the DG does not alter performance in the open field but impairs spatial, but not cued, learning/memory in the Morris water maze.** WT and $hAPP_{low}$ mice received bilateral stereotaxic injections of scr, sh1, or sh2 into the DG at 2.7–3.9 months of age. One month later, they were tested in the MWM (**a–c**) after an open-field test (**d,e**). (**a,b**) Performance of mice in a 60-s probe trial (platform removed) 24 h after hidden-platform training in the MWM (Fig. 3d). (**a**) Number of times mice crossed the original platform (Target) location vs. corresponding locations in other quadrants (Other). $^{**}P < 0.01$, $^{***}P < 0.001$ vs. WT mice injected with scr (Dunnett's test) or as indicated by brackets (paired *t*-test). ns, not significant. (**b**) Percent of time mice spent in the target

quadrant vs. the three other quadrants. *$P < 0.05$, ***$P < 0.001$ vs. chance (dotted line) by one-sample *t*-test. ns, not significant. **(c)** Learning curves of mice tested in the cued platform component of the MWM (two trials per day on two consecutive days). Bayesian modeling revealed no significant differences among groups. $n = 5–11$ mice per genotype and treatment (2 $hAPP_{low}$ mice injected with scr were excluded from the analysis because they showed floating behavior on >3 consecutive days). (**d,e**) The total distance the mice traveled in the open field (**d**) and the proportion of time mice spent in the center vs. periphery of the field (**e**) during the 15 min of testing. Two-way ANOVA revealed no significant effect of genotype or treatment. $n = 6–11$ mice per genotype and treatment. Bars represent means ± s.e.m.

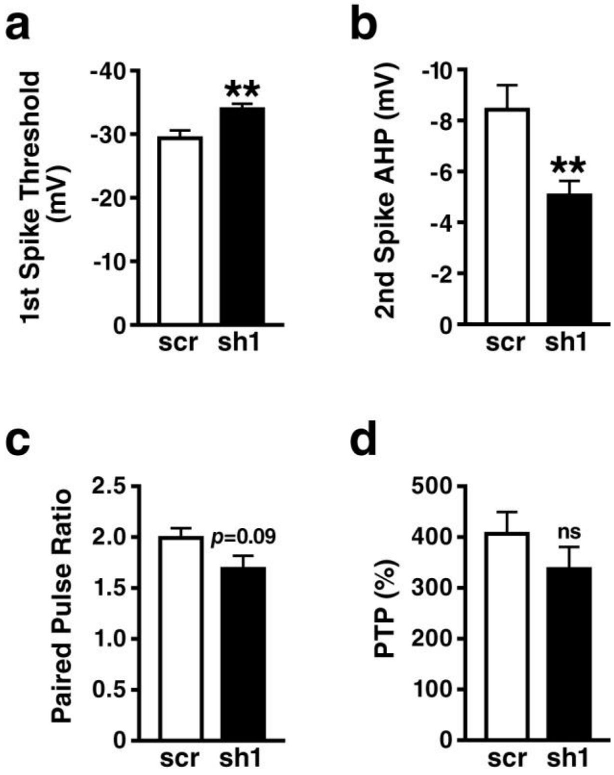

Supplementary Figure 10 | **BRCA1 Knockdown Increases Cell Excitability But Does Not Affect Short-term Plasticity.** WT mice received injections of scr or sh1 into the DG at 3 months of age. Acute brain slices were made 45–65 days later, and whole-cell or field recordings were done as in Figure 6. **(a)** Quantitation of the average first spike threshold at 500 pA stimulation. n = 32–33 cells per group (3–9 cells per mouse from 5 mice). **(b)** Quantitation of the average second spike afterhyperpolarization (AHP) at 500 pA stimulation. n = 30–31 cells per group (3–9 cells per mouse from 5 mice). **(c)** Short-term plasticity assessed by measuring the paired-pulse ratio (second pulse/first pulse) with a 50-ms interpulse interval. (**d**) Quantitation of post-tetanic potentiation (PTP; average normalized fEPSP amplitude 1–2 min after tetanic stimulation with 125 pulses at 25 Hz). n = 12 slices per group (2–3 slices per mouse from 5 mice). $^{**}P < 0.01$ vs. scr by t-test. ns, non significant. Values are means ± s.e.m.

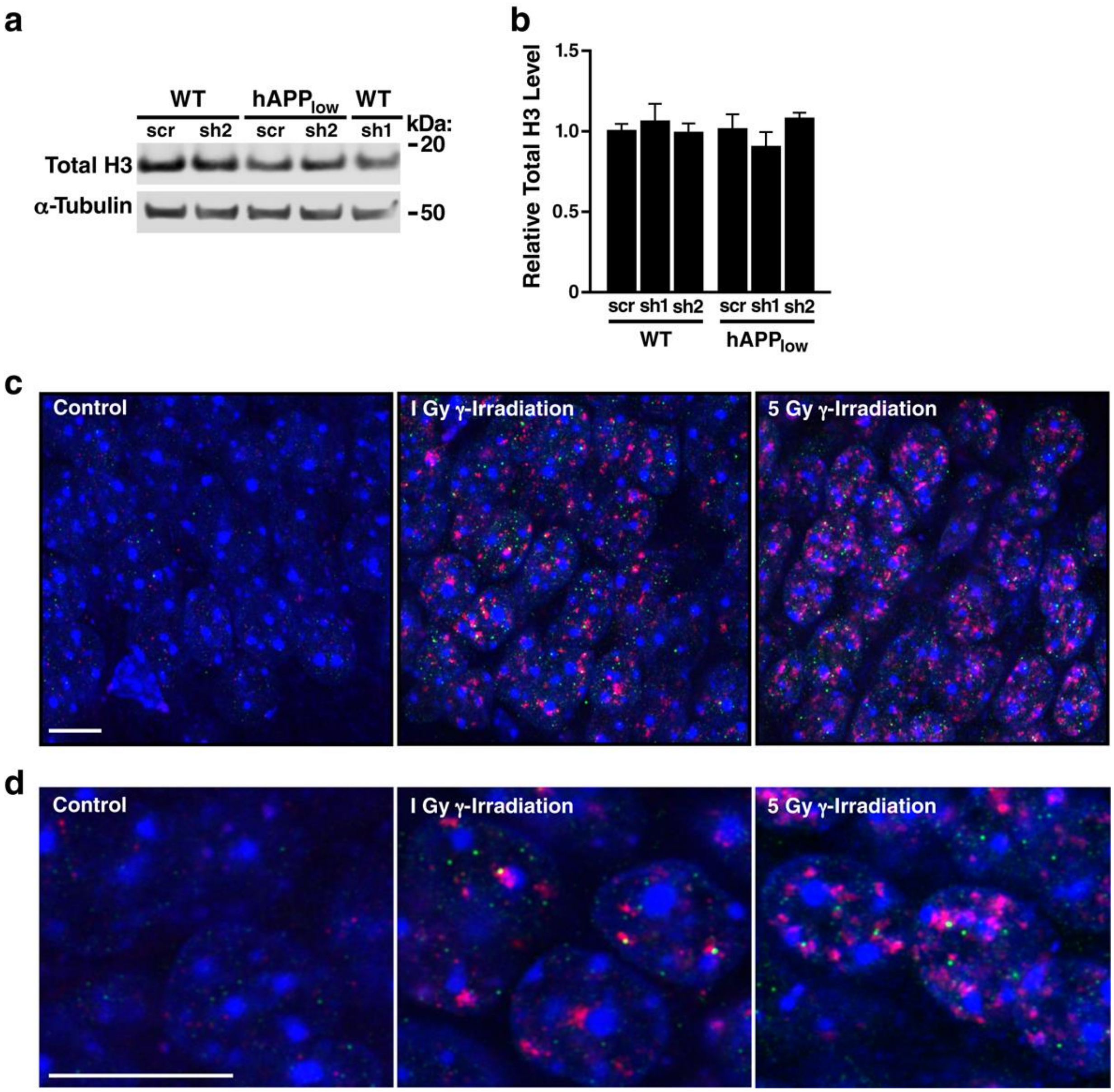

Supplementary Figure 11 | BRCA1 knockdown increases dimethylation on lysine 9 of core histone 3, an epigenetic modification that is associated with presence of DSBs. (a–b) WT and $hAPP_{low}$ mice received bilateral stereotaxic injections of scr, sh1 or sh2 into the DG at 2.7–3.9 months of age. The levels of total histone 3 in the DG were assessed 2.5 months later by western blot analysis. **(a)** Representative western blot of total histone 3. Each lane contained a sample from a different mouse. (**b**) Quantitation of western blot signals. n = 4–9 mice per genotype and treatment. The average ratio of total histone 3 to α-tubulin in scr-infected

WT mice was defined as 1.0. (**c,d**) Radiation-induced DSBs and histone modification in the DG of WT mice. After receiving 0 (control), 1, or 5 Gray (Gy) of whole-body γ-irradiation, 4–5-month-old mice were put back into their home cage for 1 h followed by histological analysis. Brain sections were immunostained for γH2A.X and Me2H3K9 and co-labeled with DAPI. **(c)** Representative Airyscan-processed confocal micrographs showing DAPI-labeled nuclear chromatin (blue), γH2A.X-positive foci (red), and Me2H3K9-positive punctae (green). Scale bar, 10 μm. **(d)** Enlarged images from the micrographs in (**c**). $*P < 0.05$ vs. leftmost bar (Bonferroni test). Bars represent means ± s.e.m.

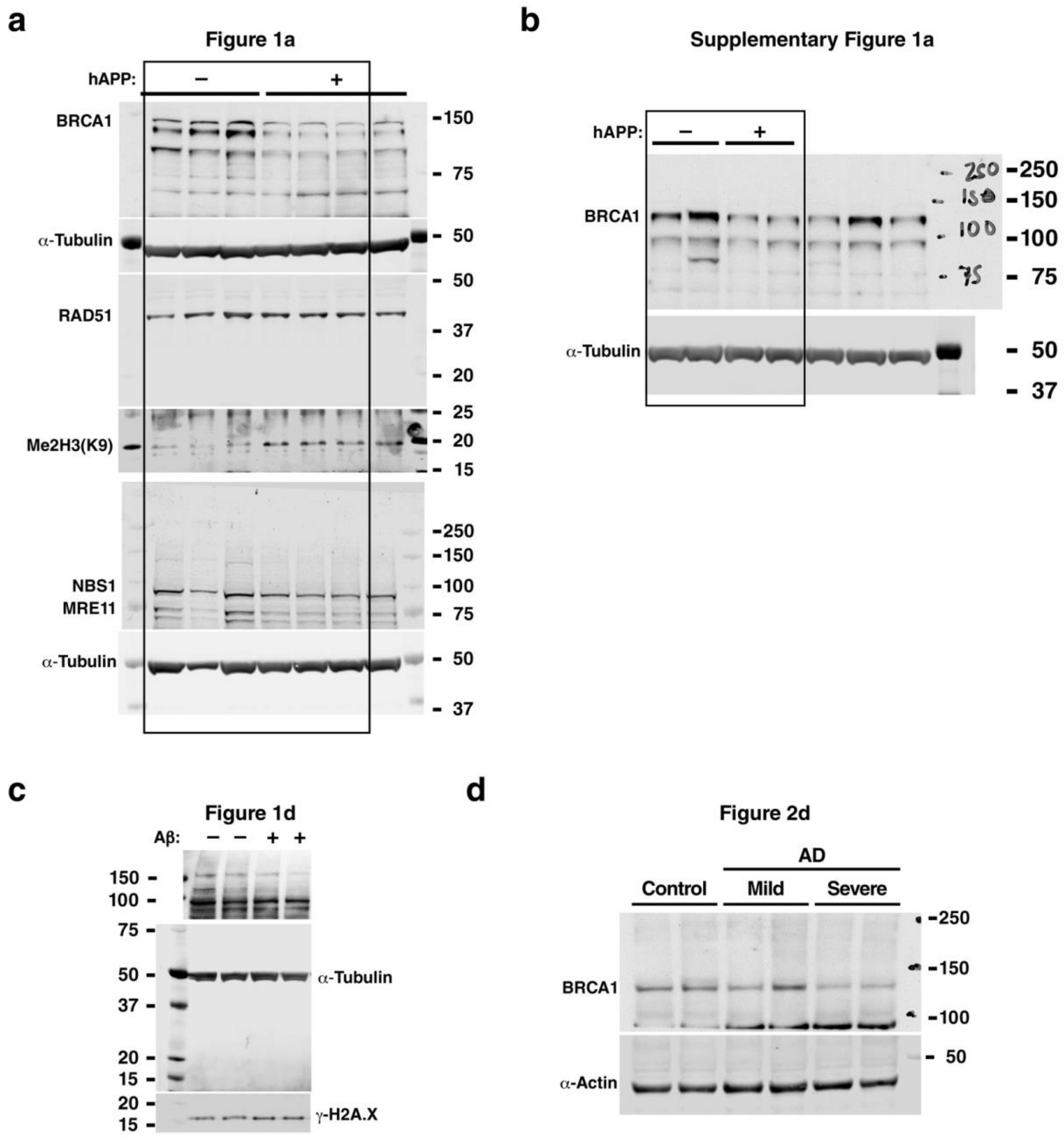

Supplementary Figure 12 | **Broader Western blot images.** Larger portions of Western blots corresponding to the components shown in **(a)** Figure 1a**, (b)** Supplementary Figure 1a, **(c)** Figure 1d, and **(d)** Figure 2d. For analysis of BRCA1 levels, membranes were cut under or at the 75 kDa weight mark, so the upper part could be stained with anti-BRCA1 primary antibody and peroxidase-conjugated secondary antibodies, while the lower part was stained with other primary antibodies and IRD-tagged secondary antibodies.

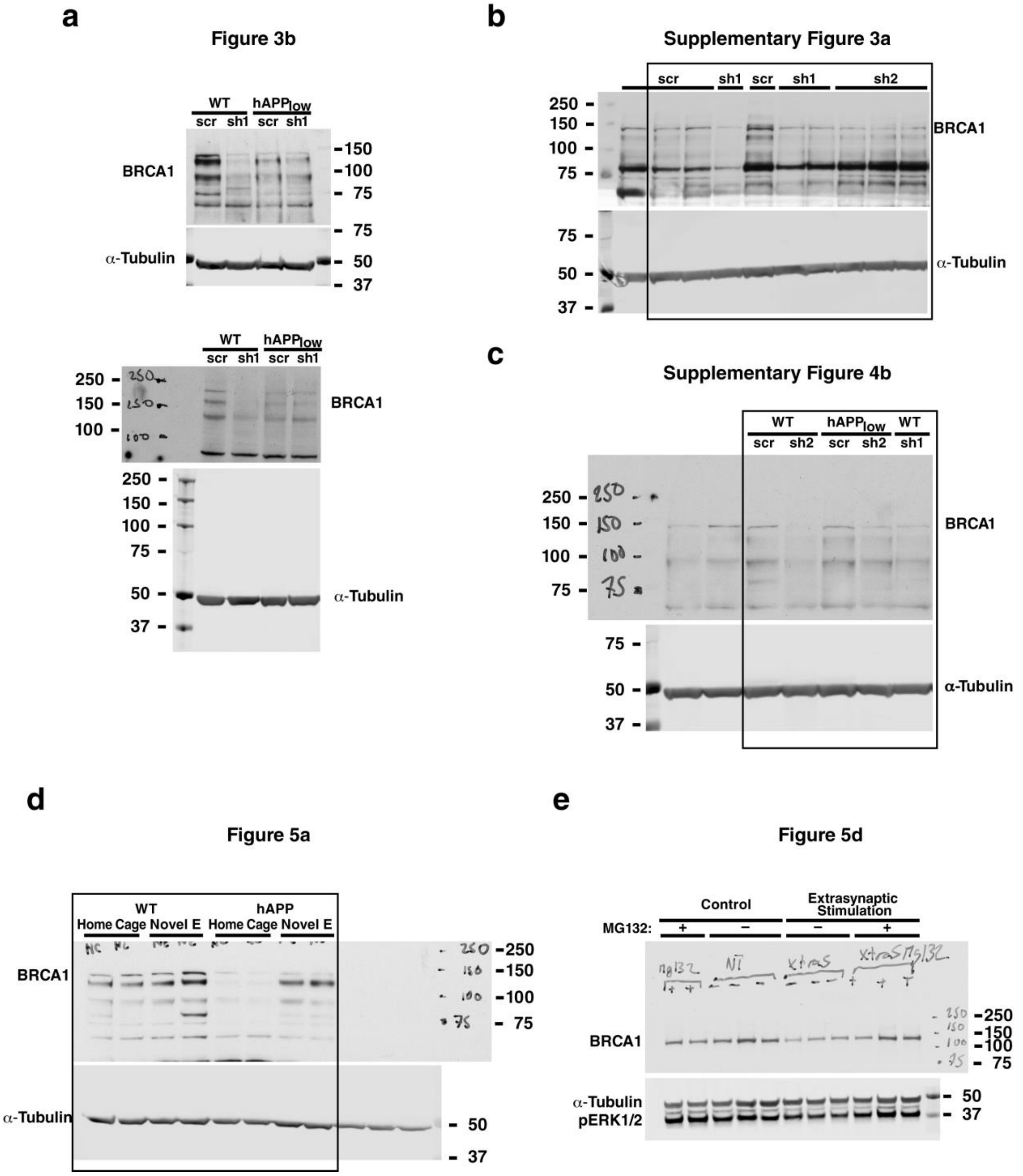

Supplementary Figure 13 | **Broader Western blot images.** Larger portions of Western blots corresponding to the components shown in **(a)** Figure 2b**, (b)** Supplementary Figure 3a, **(c)** Supplementary Figure 4b, **(d)** Figure 5a, and **(e)** Figure 5d.

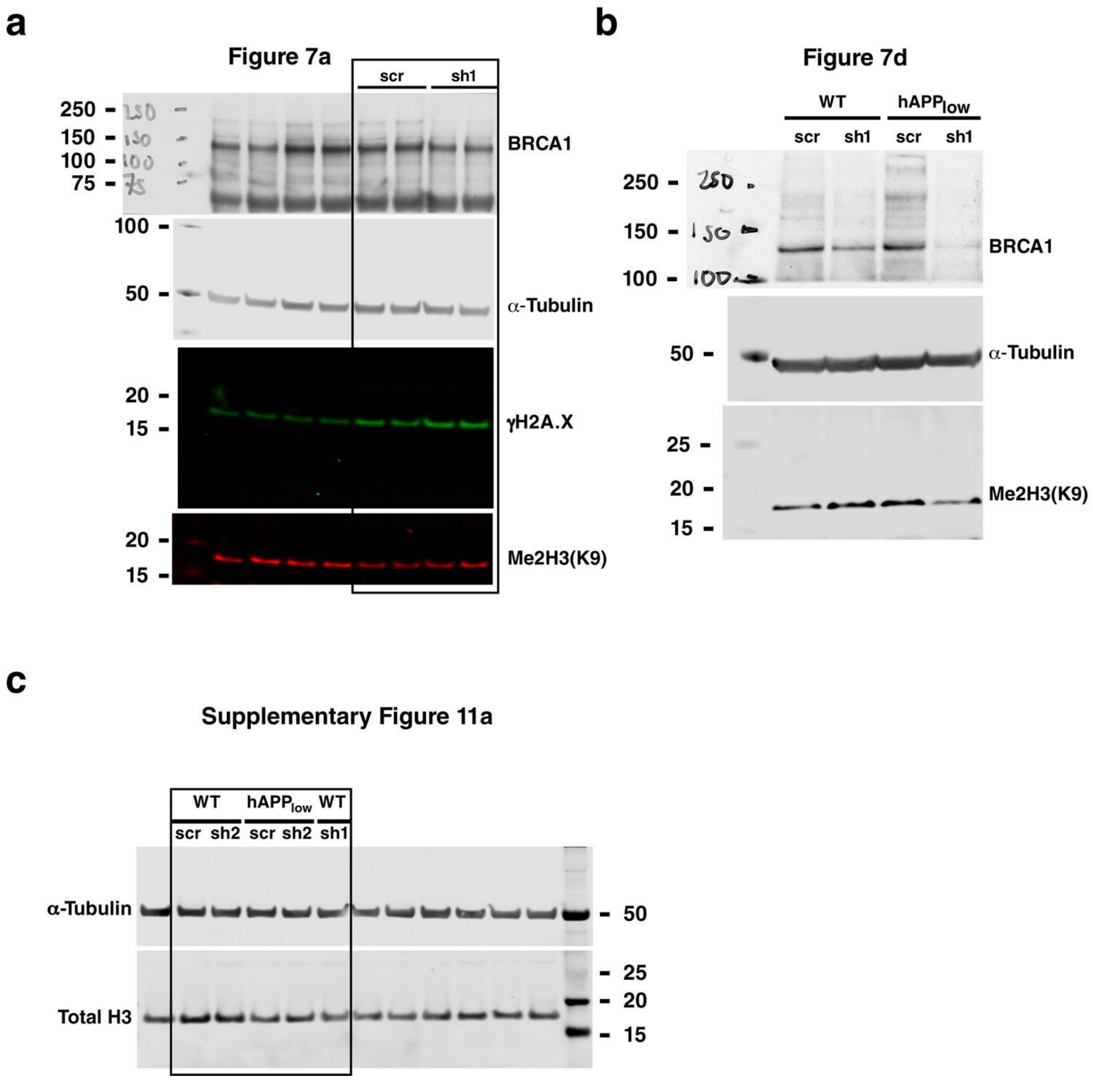

Supplementary Figure 14 | Broader Western blot images. Larger portions of Western blots corresponding to the components shown in **(a)** Figure 7a**, (b)** Figure 7d, and **(c)** Supplementary Figure 11a. Because MeH3(K9) and γH2A.X (a) are both ~17 kDa in size, their western blot signals overlap. Therefore, Me2H3(K9) was detected with a rabbit primary antibody and a 680 IRD-conjugated secondary antibody (in red) and γH2A.X protein with a mouse primary antibody and an 800 IRD-conjugated secondary antibody (in green).
